# Epromoters function as a hub to recruit key transcription factors required for the inflammatory response

**DOI:** 10.1038/s41467-021-26861-0

**Published:** 2021-11-18

**Authors:** David Santiago-Algarra, Charbel Souaid, Himanshu Singh, Lan T. M. Dao, Saadat Hussain, Alejandra Medina-Rivera, Lucia Ramirez-Navarro, Jaime A. Castro-Mondragon, Nori Sadouni, Guillaume Charbonnier, Salvatore Spicuglia

**Affiliations:** 1 Aix-Marseille University, INSERM, TAGC, UMR 1090 Marseille, France; 2 Equipe Labellisée Ligue Contre le Cancer, Paris, France; 3 Vinmec Research Institute of Stem cell and Gene technology, Vinmec Healthcare System, Hanoi, Vietnam; 4 Laboratorio Internacional de Investigación sobre el Genoma Humano, Universidad Nacional Autónoma de México, Juriquilla, Mexico; 5 Present Address: Centre for Molecular Medicine Norway (NCMM), Nordic EMBL Partnership, University of Oslo, 0318 Oslo, Norway

**Keywords:** Gene regulation, Inflammation

## Abstract

Gene expression is controlled by the involvement of gene-proximal (promoters) and distal (enhancers) regulatory elements. Our previous results demonstrated that a subset of gene promoters, termed Epromoters, work as bona fide enhancers and regulate distal gene expression. Here, we hypothesized that Epromoters play a key role in the coordination of rapid gene induction during the inflammatory response. Using a high-throughput reporter assay we explored the function of Epromoters in response to type I interferon. We find that clusters of IFNa-induced genes are frequently associated with Epromoters and that these regulatory elements preferentially recruit the STAT1/2 and IRF transcription factors and distally regulate the activation of interferon-response genes. Consistently, we identified and validated the involvement of Epromoter-containing clusters in the regulation of LPS-stimulated macrophages. Our findings suggest that Epromoters function as a local hub recruiting the key TFs required for coordinated regulation of gene clusters during the inflammatory response.

## Abstract (web summary)

Some promoters, termed Epromoters, can display enhancer activity and regulate distal genes in their endogenous context. Here the authors report the broad involvement of Epromoters in the coordinated activation of interferon (IFN) response. They show activation of a number of IFN-inducible genes is a result of Epromoters regulating expression of an entire IFN response gene cluster.

## Introduction

Regulation of gene transcription in higher eukaryotes is accomplished through the involvement of transcription start site (TSS)-proximal (promoters) and -distal (enhancers) regulatory elements. It is now well acknowledged that enhancer elements play an essential role during development and cell differentiation, while genetic alterations in these elements are a major cause of human diseases^1^. The classical definition of enhancers implies the property to activate gene expression at a distance, while promoters induce local gene expression. However, this basic dichotomy has been challenged by broad similarities between promoters and enhancers^2,3^.

While epigenomic studies allow genome-wide identification of putative enhancers, they do not provide direct proof of enhancer function or activity. To tackle this problem, several high-throughput reporter assays have been developed and implemented to study enhancer activity in different cellular contexts^4^. Using high-throughput reporter assays in different cellular contexts from drosophila to humans, it was found that a subset of gene-promoters, also termed Epromoters, displays enhancer activity when tested in vitro^5–9^. Importantly, several concomitant studies provided evidence that some core promoters indeed control distal gene expression in their natural context^5,10–12^, and that human genetic variation within Epromoters influences distal gene expression, as assessed by expression Quantitative Trait Loci (eQTL)^5,13^. Overall, the finding that a subset of promoters works also as bona fide enhancers have significant implications for the understanding of complex gene regulation in normal development and open the intriguing possibility that sequence variation lying within a subset of (E)promoters might impact on physiological traits or diseases by directly regulating distal gene expression^3^.

Previous studies have suggested a link between the Epromoter function and the stress responses, particularly the regulation of interferon-response genes^5,14,15^. The inflammatory response requires the activation of a complex transcriptional program that is both cell-type- and stimulus-specific and involves the dynamic regulation of hundreds of genes^16^. In the context of inflamed tissue, extensive changes in gene expression occur in both parenchymal cells and infiltrating cells of the immune system. Transcriptional regulation of interferon (IFN)-response genes is one of the most intensively studied regulatory processes. In general, type I (IFNa/IFNb) and II (IFNg) interferons are responsible for regulating and activating the immune response. Expression of type I interferons can be induced in virtually all cell types upon recognition of viral components, especially nucleic acids, whereas type II interferon is induced by cytokines such as IL-12, and its expression is restricted to immune cells. While most transcription factors (TFs) involved in type I and II response are well characterized (e.g., STAT1/2 and IRFs for type I), there are still open questions concerning the precise epigenetic mechanisms involved in the rapid and precise activation of IFNa-stimulated genes. For instance, not all promoters of IFNa-stimulated genes are bound by the aforementioned factors and underlying mechanisms still await further clarification^17^.

We hypothesize that Epromoters might play an important role in the coordination of rapid gene induction after IFNa stimulation, and more generally during the inflammatory response. By combining high-throughput reporter assays and gene expression analyses, we find that a significant subset of IFNa-induced genes is associated with Epromoters and regulates the induction of other neighbor genes. Consistently, we found that IFNa-induced Epromoters have the ability to specifically and efficiently recruit the interferon-response transcription factors. In fact, within a typical cluster of IFNa-response genes, the interferon response factors are found to exclusively bind to the Epromoter, which is required for the proper induction of other genes within the cluster. Predictions based on gene expression dynamics and TF-binding profiles allow us to identify and validate Epromoter-regulated clusters in primary LPS-stimulated macrophages. Thus, Epromoters have a broad role in the induction of co-regulated genes during the mammalian inflammatory response.

## Results

### Epromoters are involved in type I interferon response

To initially explore the link between Epromoters and type I interferon response, we analyzed active enhancers in HeLa cells in the presence or absence of Interferon type I response inhibitors, using published whole-genome enhancer screen from Self-Transcribing Active Regulatory Region Sequencing (STARR–seq) experiments^15^. Noted that, HeLa cells have active interferon signaling in the absence of exogenous stimulation^5,15^. The genomic distribution of active enhancers shows that the interferon-dependent enhancers (active without inhibitors) are significantly closer to the TSS (*P*-val. = 2.2 × 10^−16^; KS test) as compared to interferon-independent enhancers (actives in the presence of inhibitors) (Supplementary Fig. 1a, b). Indeed, we found that the number of proximal enhancers (<1 kb from the closest TSS; Epromoters) decreased after treatment with interferon inhibitors, while distal enhancers (>1 kb from any TSS) increased (Supplementary Fig. 1c). Interferon-dependent TSS-proximal enhancers (Epromoters) were enriched in Interferon-Stimulated Response Elements (ISRE), including binding sites for TFs of the IRF family (Supplementary Fig. 1d). In contrast, TSS-proximal enhancers in the presence of the interferon inhibitors, as well as, the TSS-distal enhancers were enriched in binding sites for developmental TFs (Supplementary Fig. 1e). This suggests that TSS-proximal enhancers (i.e., Epromoters) are preferentially activated by the type I interferon signaling in HeLa cells.

**Fig. 1:**
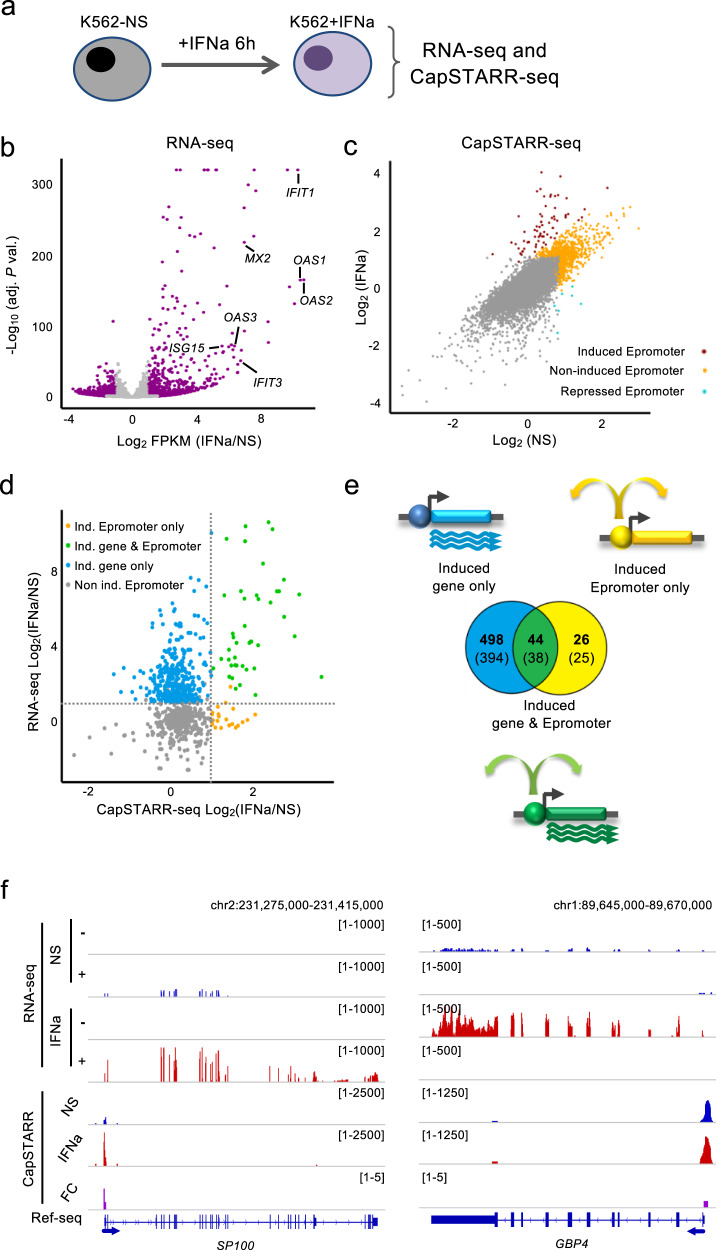
Comparison of RNA-seq and CapSTARR-seq in non-stimulated and IFNa-stimulated K562 cells. **a** Experimental approach to assess gene expression and enhancer activity of human promoters in non-stimulated (K562-NS) and IFNa-stimulated (K562 + IFNa) K562 cells. **b** Volcano plot of RNA-seq data in K562. The data plotted is the fold-change (FC) K562 + IFNa over K562-NS (log_2_ scale) of the Fragment *per* Kilobase *per* Million (FPKM) and the adjusted (adj.) *P*-value (−log_10_ scale). Genes significantly regulated (adj. *P*-val. < 0.001) are highlighted in purple. Some typical ISGs are indicated. **c** Scatter plot showing the CapSTARR-seq signal in K562-NS and K562 + IFNa. The data plotted is the CapSTARR-seq signal over the input (log_2_ scale). IFNa-induced Epromoters (brown), non-induced Epromoters (yellow), and repressed Epromoters (cyan) are highlighted. **d** Scatter-plot of RNA-seq and CapSTARR-seq signals, displaying only IFNa-induced genes or genes harboring an Epromoter. The data plotted is the RNA-seq and the CapSTARR-seq fold-change of K562 + IFNa over K562-NS (log_2_ scale). The different categories of genes in function of the IFNa response are indicated. Dot lines indicated a fold-change of 2. **e** Venn diagram showing the overlap between significantly induced genes and induced Epromoters. The number of promoters (bold) and the associated genes (under brackets) is shown. Note that some genes can be associated with multiple categories due to the presence of alternative promoters. **f** Examples of IFNa-induced genes with induced and non-induced Epromoters. The genomic tracks show the RNA-seq and CapSTARR-seq signal in non-stimulated (blue), IFNa-stimulated (red) K562 cells and the fold-change (purple). Positive and negative signs indicate the RNA-seq signal on the sense and anti-sense strands, respectively. Arrows indicate the orientation of the gene transcripts.

To directly assess the contribution of Epromoters to the regulation of type I interferon response, we performed paralleled experiments to assess gene expression (RNA-seq) and enhancer activity of gene promoters (CapSTARR-seq) (Fig. 1a and Supplementary Data 1) in the K562 cell line with and without IFNa stimulation. CapSTARR-seq, is a high-throughput reporter assay, coupling capture of regions of interest to STARR-seq^18,19^ and was previously used to identify Epromoters in unstimulated K562 cells^5^. Notably, the K562 cells do not express type I interferon response genes in the absence of stimulation and do not induce an interferon response after DNA transfection^5,15^, making this cell line an appropriate model to study type I interferon stimulation. To quantify the enhancer activity of gene promoters, we used a capture-based library containing 17,941 promoters (−200 bp to +50 bp from the TSS), corresponding to 14,188 RefSeq-defined coding genes as previously described^5^. Analyses of RNA-seq after IFNa stimulation of K562 cells resulted in 426 induced and 436 repressed genes (*P*-val. < 0.001; Fig. 1b). However, induced genes generally reached higher significance as compared to repressed genes, consistent with previous findings^20^. Highly IFNa-Induced genes included classical type I interferon response genes such as the *MX*, *OAS*, and *IFIT* family of genes (Supplementary Data 1).

The enhancer activity was calculated as the fold-change of the CapSTARR-seq signal over the input library and was highly reproducible between replicates (Pearson’s R^2^ > 0.9; Supplementary Fig. 2a). Gene promoters were defined as Epromoter when the enhancer activity was above the inflection point of the ranked signal of all captured regions, as previously described^5^. Induced Epromoters were defined as Epromoters that gained enhancer activity by a fold-change of 2 between IFNa-stimulated and non-stimulated K562 cells. We identified 429 constitutive Epromoters, as well as 70 induced and 7 repressed Epromoters (Fig. 1c, Supplementary Data 1). Combining the RNA-seq and CapSTARR-seq experiments allowed us to define three sets of IFNa-response loci in K562 cells (Fig. 1d, e and Supplementary Data 2): (i) 498 promoters associated with 394 IFNa-induced genes but without induced Epromoter activity (induced gene only), (ii) 44 promoters with induced Epromoter activity and associated with 38 IFNa-induced genes (induced gene & Epromoter), and (iii) 26 promoters associated with IFNa-induced Epromoter activity and associated with 25 non-induced genes (induced Epromoter only). Moreover, from the 394 induced gene only, 16 were associated with a constitutive Epromoter (Supplementary Data 1). Of note, the set of “induced gene & Epromoter” displayed stronger induction than the set of “induced gene only” (Fig. 1d and Supplementary Fig. 2b). Examples of “induced gene & Epromoter” (*SP100*) and “induced gene only” (*GBP4*) loci are shown in Fig. 1f. Taken together these results suggest a potential involvement of Epromoters in the type I interferon response.

### IFNa-induced Epromoters specifically recruit the interferon response factors

**Fig. 2:**
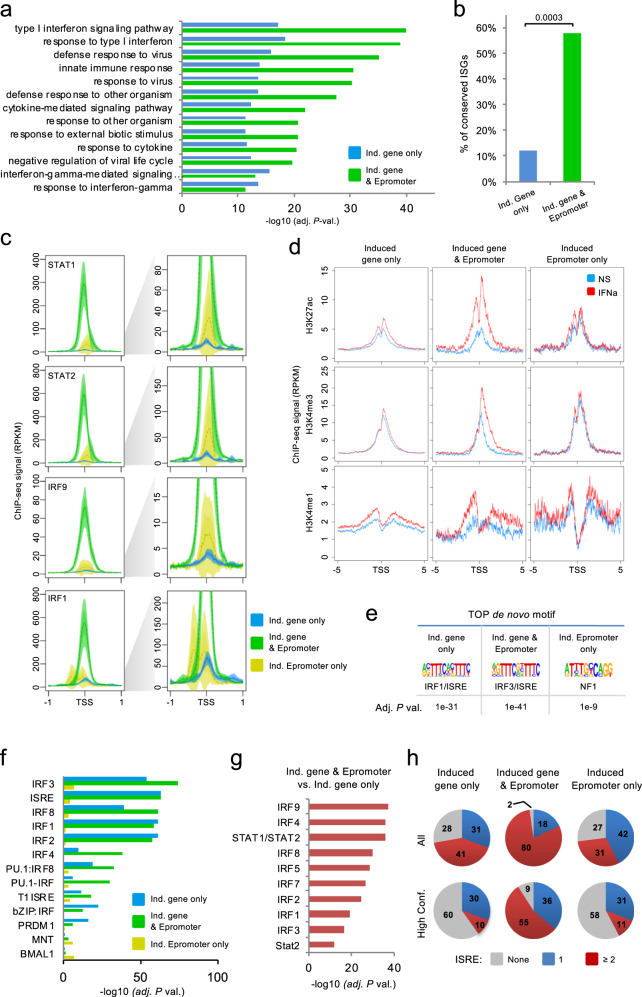
Genomic and epigenomic characteristics of IFNa-induced genes and Epromoters. **a** Top10 Gene Ontology enrichment for the biological process of genes associated with “induced gene only” (blue) and “induced Epromoter & gene expression” (green). No enrichment was found in the “induced Epromoter only” category. Data plotted are the adjusted *P*-value (−log_10_ scale) of the enrichment. **b** Percentage of conserved ISGs^21^ found in the “induced gene only” and “induced gene & Epromoter” sets. Chi-square test was performed and *P*-values are annotated. **c** Average profiles of ChIP-seq signals for the TFs STAT1, STAT2, IRF9, and IRF1 in K562 cells stimulated with IFNa for 6 h. The groups shown were defined in Fig. 1e. The solid line represents the mean of the signal while the colored area represents the 95% confidence interval. The left panels display the average profiles set at the maximum scale, while the right panels display the same profiles set at a lower scale. **d** Average profiles of ChIP-seq signals for the histone modifications, H3K27ac, H3K4me3 and H3K4me1 in K562 cells non-stimulated (blue) or stimulated with IFNa for 6 h (red). The solid line represents the mean of the signal. **e** Top de novo motifs found enriched in “induced gene only”, “induced gene & Epromoter” and “induced Epromoter only” sets, using the HOMER tool^73^. The enrichment adjusted *P*-value is shown. **f** Motif enrichment analysis in promoter regions from the groups defined in Fig. 1E using the HOMER tool. Only the top 10 motifs for each promoter set are shown. The data shown is the adjusted *P*-value (−log_10_). **g** Comparison of the relative number of binding sites per promoter between the “induced Epromoter and induced gene” versus the “induced genes only” sets. The data shown is the adjusted *P*-value (−log_10_) of the comparison. **h** Pie-charts showing the percentage of promoters from the indicated datasets that contain either none (gray), one (blue) or two or more ISRE-binding sites (red) (merged sites for IRF1–9 and STA1-2 motifs) based on the Jaspar 2020 annotation^74^ by default (All ISRE sites) or with a *P*-value < 1e-4 (High confidence).

To better understand the relevance of the three sets of IFNa-stimulated loci, we analyzed the functional enrichment of the associated genes. As expected, the set of “induced gene only” was primarily linked to type I interferon response and related pathways. However, the set of “induced gene & Epromoter” achieved higher enrichment in those pathways even though they represent a minority (9%) of IFNa-response genes (Fig. 2a and Supplementary Data 3). The set of “induced Epromoter only” was not associated with any biological process, likely due to the low number of genes. However, manual inspection of the GO biological processes reveals three genes associated with the interferon response (*HLA-G*, *MVB12A*, *TRIM34*; Supplementary Data 3). Besides, analyses of a dataset of interferon-stimulated genes (ISGs) across different animal species^21^, shows that 58% (22) of the genes from the set of “induced gene & Epromoter” overlapped with a conserved set of 98 ISGs, as compared with 12% (47 genes) of the “induced gene only” set (Fig. 2b; *P*-val. = 0.00001, Chi-Square test), suggesting that induced Epromoters are preferentially associated with a conserved interferon response.

Classical type I interferon response requires binding to the ISRE by the regulatory complex formed by phosphorylated STAT1 and STAT2 TFs with the constitutive IRF9 TF also referred to as the ISGF3 complex^16,22^. Subsequent activation can be also mediated by the inducible IRF1 TF. To assess the binding of these TFs to the three sets of IFNa-response promoters, we analyzed previously generated ChIP-seq data in IFNa-stimulated K562 cells for STAT1, STAT2, and IRF1 (Supplementary Data 4) and generated ChIP-seq for IRF9. Strikingly, all four TFs preferentially bound to the set of “induced gene & Epromoter” as compared to the other two sets. In particular, the set of promoters associated with “induced gene only” displayed binding levels close to the background (Fig. 2c). Recruitment of these TFs was specific to IFNa-induced Epromoters as no binding was observed in non-induced Epromoters (Supplementary Fig. 2c). As expected, the majority of induced Epromoters were bound by the ISGF3 complex and were more likely to be associated with a conserved ISGs (Supplementary Fig. 2d; *P*-val. = 0.0002; Chi-Square test).

To gain insight into the epigenetic dynamic of these promoters, we performed ChIP-seq for the histone modifications H3K4me3, H3K27ac and H3K4me1 in non-stimulated and IFNa-stimulated K562 cells (Fig. 2d and Supplementary Data 4). For all three histone modifications, the strongest gain of the signal was observed for the set of “induced gene & Epromoter”. Thus, the set of “induced gene & Epromoter” has the ability to recruit the key TFs associated with the interferon response and acquires a highly active chromatin state, likely associated with their enhancer activity.

To determine whether the preferential binding of IFN-response TFs to the induced Epromoters was due to an intrinsic feature of the DNA sequence we analyzed the enrichment in TFBS in the three groups of promoters. De novo discovery of DNA motifs (Fig. 2e) as well as global enrichment analyses of known motifs (Fig. 2f), demonstrated that promoters associated with the “induced gene only” and “induced gene & Epromoter” sets, but not with the “induced Epromoter only” set, were similarly enriched in STAT- and IRF-binding sites and generally contain the consensus ISRE. Therefore, the presence of STAT/IRF-binding sites did not explain per se the higher efficiency of TF recruitment observed at induced Epromoters.

Differential enrichment analysis of ISRE-associated binding sites shows that the STAT and IRF motifs were significantly enriched in the “induced gene & Epromoter” set as compared with the “induced gene only” set (Fig. 2g). To assess whether the difference in the density of TFs could be different between the three promoter sets, we first computed the number of sites *per* promoter found for each IRF factor (Supplementary Fig. 3). We observed that the density of IRF sites per promoter was significantly higher in the “induced gene & Epromoter” set as compared with the two other sets.

Next, we computed the number of non-redundant ISRE sites *per* promoter by combining all STAT1-2 and IRF1-9 sites (Fig. 2h, top panels). The “induced gene & Epromoter” set harbor a majority of promoters with 2 or more ISRE sites (80%; 35). In contrast, only 41% (207) and 31% (8) of “induced gene only” and “induced Epromoter only” sets, respectively, displayed 2 or more ISRE sites. The same analysis performed with high confidence sites shows that the majority of the sites found in the promoters of the “induced gene only” and “induced Epromoter only” sets are of lower affinity (Fig. 2h, bottom panels). While 55% (24) of promoters of the “induced gene & Epromoter” set are associated with 2 or more high confidence ISRE sites, only 10% (48) and 11% (3) of the other two sets of promoters contains 2 or more high confidence ISRE sites. This suggests that both the high density and the quality of ISRE sites likely contribute to the efficient recruitment of the STAT1/2 and IRFs complexes and that this is a specific property of the IFNa-stimulated Epromoters.

### Clusters of IFNa-induced genes are regulated by Epromoters

**Fig. 3:**
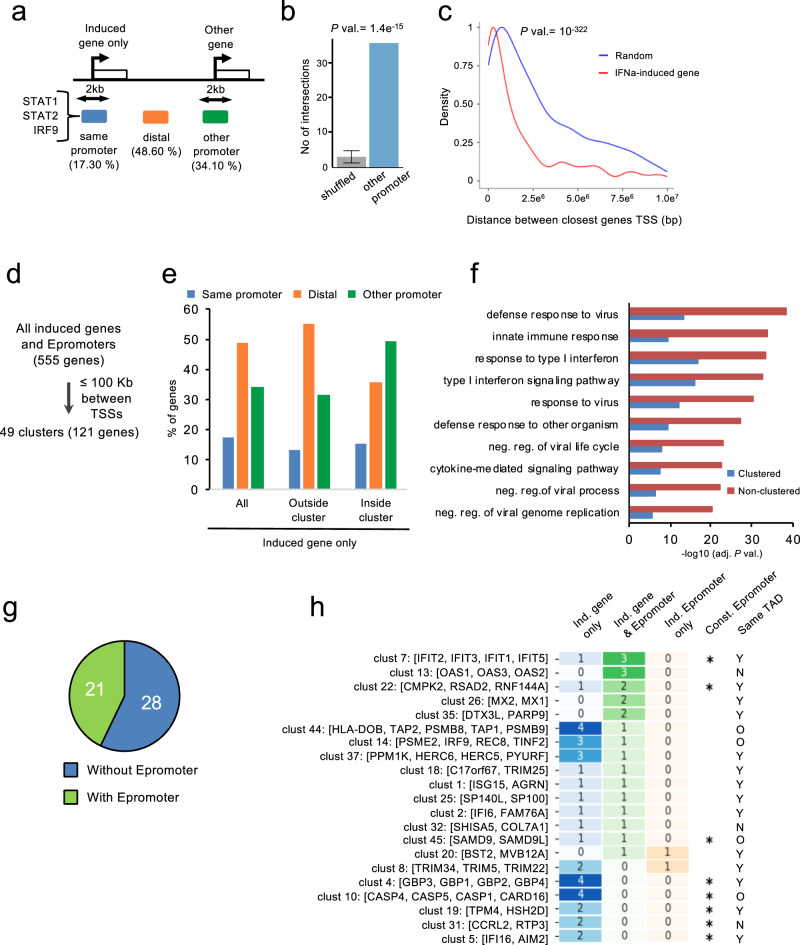
Clustering of IFNa-induced Epromoters and genes. **a** Schematic diagram showing the percentage of the closest ISGF3 binding with respect to the TSS of the “induced gene only” set. ISGF3-binding peaks in the same promoter (blue; ≤1 Kb from the TSS of “induced gene only”; another promoter (green; ≤1 Kb from the TSS of other genes), or intergenic region (orange; >1 Kb from any TSS of a coding gene). **b** Assessment of the significance of ISGF3 binding in other gene promoters (from Fig. 3a, green) by the OLOGRAM tool^76^, the shuffled plot represents the mean and s.d. of 100 random iterations. The observed number of intersections is shown in blue and the mean of 100 shuffled regions are shown in gray. Error bars represent the standard deviation of the shuffled distribution. Statistical significance was calculated against a negative binomial model. **c** The density distribution of the distance between pairs of closest genes using the list of IFNa-induced genes (red) or a set of the same number of randomly selected genes (blue) from the human genome. **d** Summarized diagram of the clustering pipeline of all induced genes and Epromoter-associated genes from which the distance between the TSS was less than 100 Kb was considered to belong to the same cluster. **e** Percentage of the closest ISGF3 binding with respect to the TSS of the “induced gene only” set, inside and outside a cluster (<100 Kb). **f** Top 10 enriched Gene Ontology biological process associated with the “induced gene only” sets that are clustered (blue) or non-clustered (red). Data plotted are the adjusted *P*-value (-log_10_ scale) of the enrichment. **g** Pie-chart with the number of clusters that contain (green) or not (blue) at least one Epromoter. The detailed list of clusters is provided in Supplementary Data 5. **h** Gene clusters harboring at least one Epromoter. The data shown contain the cluster number and the genes within the cluster. The heatmap shows the number of genes in each category. The induced gene (blue), induced gene & Epromoter (green), and induced Epromoter only (orange) groups are illustrated. The number of genes for each category is indicated and their value is represented by color intensity. The presence of constitutive Epromoters in the clusters is shown by a star (*). Whether the clustered genes were found within the same Topological Associated Domain (TAD) (Y), different TADs (N), or outside any TAD (O) is indicated.

The above results showed that the majority of IFNa-responding genes do not efficiently recruit the key IFNa-response TFs. One potential explanation is that the induction of these genes requires the action of distal regulatory elements, which might involve either typical enhancers or Epromoters. We reasoned that for induction to take place, a given locus needs to be associated with a regulatory element recruiting the ISGF3 complex (i.e., STAT1/STAT2/IRF9). The IFN-response element might be located at the promoter of the same gene or at another regulatory element, which might overlap (Epromoter-like) or not (TSS-distal or “typical” enhancers) the promoter of another gene. To address this issue, we determined the distance of the closest binding of the ISGF3 complex (i.e., overlapping STAT1/STAT2/IRF9 ChIP-seq peaks) to the promoter of all “induced genes only” set and classified it as located within the same promoter, in a TSS-distal region or another promoter (Fig. 3a). As expected only a minority of the promoters associated with the “induced genes only” loci (17.30 %) recruited the ISGF3 complex. For 48.60 %, the closest ISGF3 binding was located in a TSS-distal region, indicating that half of the induced genes might be regulated by typical enhancers. However, for more than one-third of induced genes loci (34.10 %), the closest ISGF3 peak was found in another promoter. Importantly, the binding of the ISGF3 complex to a distal promoter was much more frequent than expected by chance (Fig. 3b). To further compare the potential regulation by intergenic and TSS-proximal (either same o other promoters) regions, we analyzed the ChIP-seq profiles of TFs and histone modifications. All three sets of ISGF3-binding regions bound similar levels of IFN-responsive factors (Supplementary Fig. 4a), consistent with the presence of a composite ISGF3 peak. The ISGF3-binding regions displayed increased histone modification levels after IFNa stimulation (Supplementary Fig. 4b). As expected, intergenic ISGF3-bound regions were associated with an enhancer-like chromatin signature (high H3K27ac and H3K4me1), while TSS-proximal ISGF3-bound regions were associated with a promoter-like signature (high H3K27ac and H3K4me3). As described above for the induced Epromoters, the majority of both distal and proximal ISGF3-bound regions contains more than one ISRE site (Supplementary Fig. 4c).

Given the significant frequency of induced genes associated with an ISGF3 complex located in the promoter of another gene, we hypothesized that many induced genes might be located in clusters, likely sharing the same IFNa-stimulated regulatory elements. Therefore, we analyzed the genomic distance between all IFNa-induced genes. We found that induced genes were located closer to each other than expected by chance (Fig. 3c; *P*-val. = 10^−322^; KS test), and frequently to less than 100 kb from a constitutive or induced Epromoter (Supplementary Fig. 5a), suggesting that a subset of IFNa-induced genes are located in clusters and might be co-regulated by Epromoters.

Based on the above observations, we aimed to identify clusters of induced genes and Epromoters for which the corresponding TSS are located less than 100 kb from each other (Fig. 3d). We identified a total of 49 clusters encompassing 121 IFNa-induced loci (Supplementary Data 5). Of 286 “induced gene only” outside clusters, 158 (55.3%) were associated with an ISGF3 complex located in an intergenic region and 90 (31.5%) in another promoter (Fig. 3e). In contrast, of 73 “induced gene only” within clusters, 26 (35.6%) were associated with an ISGF3 complex located in an intergenic region and 36 (49.3%) in another promoter (Fig. 3e). Thus, induced genes within clusters appeared to be preferentially regulated by Epromoters, as compared to induced genes outside clusters (*P*-val. = 0.001, Chi-Square test). Moreover, clustered genes were more enriched in interferon-related biological processes as compared to the unclustered genes (Fig. 3f).

We further explored the regulation of clusters of induced genes by Epromoters. We found that 21 clusters (42%) contain either constitutive or induced Epromoters (Fig. 3g) and were generally located within the same Topological Associated Domain (TAD) in K562 cells (Fig. 3h). Among these clusters, we found typical IFNa-response gene families, such as *OAS*, *IFIT, MX*, and *TRIM* (Fig. 3h). The lack of association with Epromoters for the remaining 28 clusters might indicate either a regulation by a typical enhancer or the presence of an Epromoter that was not detected based on our relatively stringent criteria to select induced genes and Epromoters. For instance, the APOBEC3 locus was found to contain three induced genes (*APOBEC3D*, *APOBEC3F*, and *APOBEC3G*), however, it also contains the *APOBEC3C* gene, which has an Epromoter, but the differential expression was slightly under the applied threshold (Supplementary Fig. 5b).

**Fig. 4:**
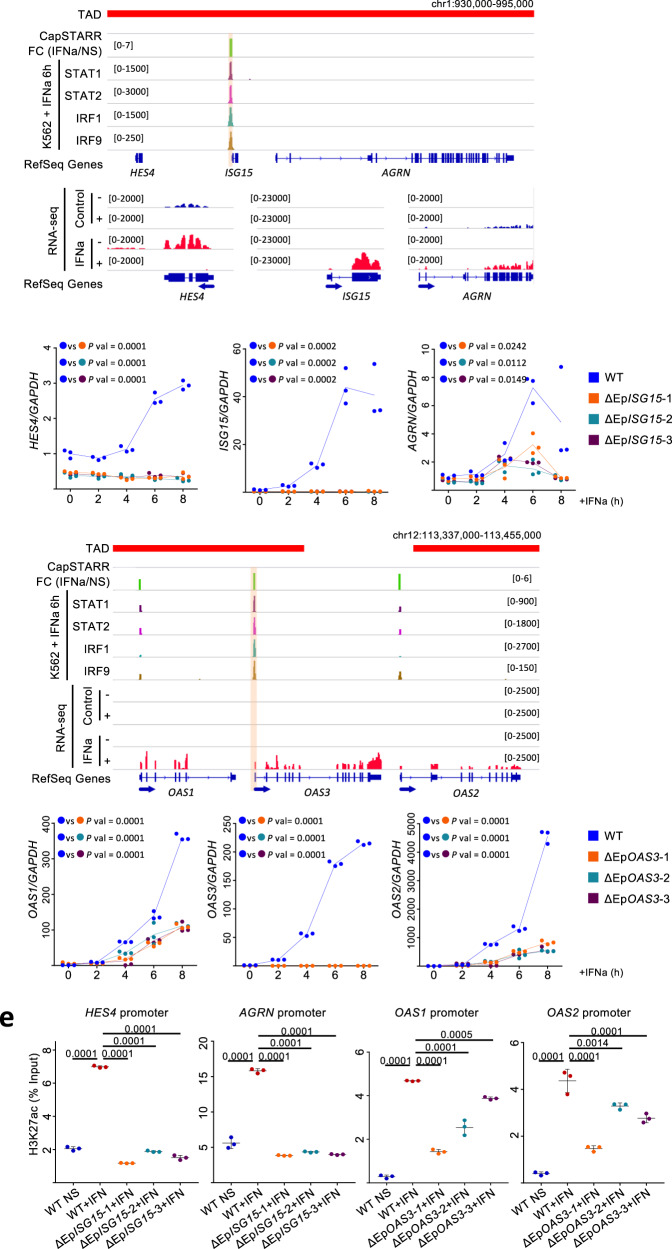
Genomic visualization and kinetic analysis of IFNa-induced genes clustered with induced Epromoters. **a**, **c** Genomic tracks centered on the *HES4*/*ISG15*/*AGRN* (**a**) and *OAS1*/*OAS2*/OAS3 (**c**) loci. Top panels show the TAD, CapSTARR-seq signal fold-change (IFNa over non-stimulated), and the ChIP-seq signal for the indicated TFs after IFNa induction. The bottom panels show the RNA-seq signals of the induced genes. Positive and negative signs indicate the RNA-seq signal on the sense and anti-sense strands, respectively. Arrows indicate the orientation of the gene transcripts. The induced Epromoter of the *ISG15* and *OAS3* genes are highlighted. **b**, **d** qPCR analysis of gene expression of the indicated genes in K562 *wild-type* (*n* = 3) and ΔEp*ISG15* (**b**, *n* = 3) or ΔEp*OAS3* (**d**, *n* = 3) mutants in non-stimulated conditions or after the indicated times of IFNa stimulation. Values represent the relative expression levels as compared to the unstimulated conditions and normalized by the *GAPDH* housekeeping gene in independent experiments. Two-side ANOVA test was performed between the *wild-type* and each of the mutant clones. **e** H3K27ac ChIP-qPCR analyses in unstimulated and IFNa-stimulated (6 h) *wild-type* K562 cells as well as stimulated ΔEp*ISG15* and ΔEp*OAS3* clones. The promoter regions of the Epromoter-regulated genes were analyzed. Values represent the percentage of input measured in ChIP replicates (*n* = 3). The error bars represent the s.d.. Two-sided Student’s *t*-test was performed between the unstimulated and stimulated wild-type cells and between stimulated wild-type cells and each of the mutant clones.

Overall, our finding raises the possibility that a subset of induced genes might be co-regulated by Epromoters located in the same cluster. Consistent with the average patterns of TF binding, visual inspection of several clusters indicated that IFN-response TFs bind preferentially to the induced Epromoters (Fig. 4 and Supplementary Figs. 6 and 7). This is the case of the *ISG15/AGRN/HES4* cluster (Fig. 4a). In this cluster, all three genes are induced by IFNa stimulation, but only *ISG15* is associated with an inducible Epromoter. *ISG15* encodes for an IFN-induced ubiquitin-like protein and plays a central role in the host antiviral response^23^. *HES4* and *AGRN* are also classified as interferon-stimulated genes (ISG) in the Interferome database^24^, while *HES4*, encoding for a bHLH (basic helix loop helix) TF, have been suggested to play a role in the IFN response^25,26^. As shown in Fig. 4a, the induced *ISG15* Epromoter, recruits the TFs STAT1, STAT2, IRF1, and IRF9 in IFNa-stimulated K562 cells, while the other induced genes located in the same cluster, *AGRN*, and *HES4*, do not. The three genes are located in the same TAD and no other region, apart from the *ISG15* Epromoter, was found to bind the TFs within the TAD (Supplementary Fig. 6, upper panel). This suggested that the *ISG15* Epromoter might harbor the necessary TFs to induce the transcription of all three genes in an Epromoter-dependent manner. To test this hypothesis, we generated three K562 clones in which the *ISG15* Epromoter has been deleted from all alleles (Supplementary Fig. 7a) and analyzed the gene expression of associated genes after different times of IFNa stimulation (Fig. 4b). As expected, the expression of the *ISG5* gene was completely abolished. Strikingly, the induction of *HES4* was completely abolished while *AGRN* expression was mostly affected at the induction peak (6 h). Although the *AGRN* and *HES4* genes have distinct stimulatory kinetics, the induction of both genes was significantly impaired in ΔEp*ISG15* clones as compared to WT K562 cells. Thus, the *ISG15* Epromoter is required for the accurate induction of the two neighbors’ IFNa-responsive genes.

We observed that some of the IFNa-induced clusters contained more than one gene associated with an Epromoter (Fig. 3d). One example is provided by the *IFIT* locus, harboring a family of genes encoding for IFN-induced proteins with tetratricopeptide repeats and have broad-spectrum activity against replication, spread, and disease pathogenesis of a range of human viruses^27^. The cluster contains four IFNa-induced genes; *IFIT3*, *IFIT1*, and *IFIT5* have inducible Epromoters that recruit all four TFs, while *IFIT2* does not have an Epromoter and only recruit the IRF1 factor (Supplementary Fig. 7c; note that *IFIT3* contains two inducible Epromoters, with the most upstream *IFIT3* promoter strongly binding all four IFN-response TFs). All five genes were located in the same TAD (Supplementary Fig. 6, middle panel). We initially generated two K562 clones where the most upstream Epromoter was deleted from all alleles (Supplementary Fig. 7b). The deletion of the upstream *IFIT3* Epromoter resulted in a moderated reduction of *IFIT3* induction. We also observed a significant impairment of *IFIT2* induction at the earliest (2 h) time point (Supplementary Fig. 7d), while the induction of *IFIT1* and *IFIT5* slightly increased. Thus, proper induction of *IFIT2* requires the function of at least one induced Epromoter within the cluster. One reason to explain the moderate impact of *IFIT3* Epromoter on *IFIT2* expression would be the synergistic interaction with the internal *IFIT3* Epromoters. Concomitant deletion of the two *IFIT3* Epromoters in two independent K562 clones, completely abolished *IFIT3* expression (Supplementary Fig. 7d). The severity of the effect on *IFIT2* induction varied between the two clones with the double *IFIT3* Epromoter deletion, but in both cases the strongest effect was observed at 2 h after IFNa treatment, consistent with the results obtained with the deletion of the upstream *IFIT3* Epromoter. Also consistent with the single deletions, the induction of *IFIT1* and *IFIT5* significantly increased in the double mutant clones. Therefore, our results suggest that *IFIT3* Epromoter(s) are required for the timely induction of *IFIT2*.

Another noticeable example is seen in the *OAS* locus, harboring three related genes encoding oligoadenylate synthase (OAS) family proteins, and playing a crucial antiviral function^28^. In this cluster, all three IFNa-induced genes were associated with inducible Epromoters (Fig. 4c). The cluster was divided into two TADs in non-stimulated K562 cells (Supplementary Fig. 6, lower panel). However, visual inspection of the Hi-C matrix suggested that the insulation at the TAD boundary within the *OAS* cluster in non-stimulated cells is very weak. We noticed that the *OAS3*-associated Epromoter displayed a higher binding of the four interferon-response TFs, as compared with the *OAS1* and *OAS2* Epromoters. Therefore, we asked whether the specific binding of IFN-response factors was a predictor of the distal regulatory activity of the Epromoter. To explore the specific contribution of each Epromoter towards the regulation of the entire cluster, we generated K562 clones where each of the *OAS* Epromoters have been deleted from all alleles (Supplementary Fig. 8a). The deletion of *OAS1* and *OAS2* Epromoters did not affect the induction of *OAS3* expression (Supplementary Fig. 8b). However, the deletion of the *OAS3* Epromoter resulted in a dramatic reduction of *OAS1* and *OAS2* induction after IFNa stimulation (Fig. 4d). To control for potential bias that might be induced by CRISPR-mediated homologous recombination, we analyzed the induction of *OAS1* and *OAS2* genes after IFNa stimulation from the pool of K562 cells individually transfected with each of the two sgRNAs used for Ep*OAS3* deletion or a clone with an inert insertion within the first intron of *OAS3*. In all three situations, the induction of the three *OAS* genes was not affected (Supplemental Fig. 8c). Finally, the rescue of *OAS3* expression in ΔEp*OAS3* clones did not affect *OAS1* nor *OAS2* induction levels (Supplemental Fig. 8d), indicating direct regulation of neighboring gene expression by the *OAS3* Epromoter. Overall, these results suggested that within a cluster of IFN-induced genes only the promoters that efficiently recruit the key IFN-response factors have the capability to provide a distal regulatory function.

Finally, we explored whether the deletion of the Epromoters also have an impact on the neighbor promoters at the chromatin level. To this end, we analyzed the level of H3K27ac at the *ISG15* and *OAS3* Epromoter-associated promoters by ChIP (Fig. 4e). Deletion of the *ISG15* Epromoter completely abolished the gain of H3K27ac at the *HES4* and *AGRN* promoters observed in wild-type K562 cells after 6 h of IFNa treatment. Deletion of the *OAS3* Epromoter resulted in a less severe, although significant, impairment of H3K27ac after IFNa stimulation. Thus, Epromoters might be required for the chromatin remodeling required for the induction of the IFNa-response associated genes.

### Promoter versus enhancer activity of the *OAS3* Epromoter

**Fig. 5:**
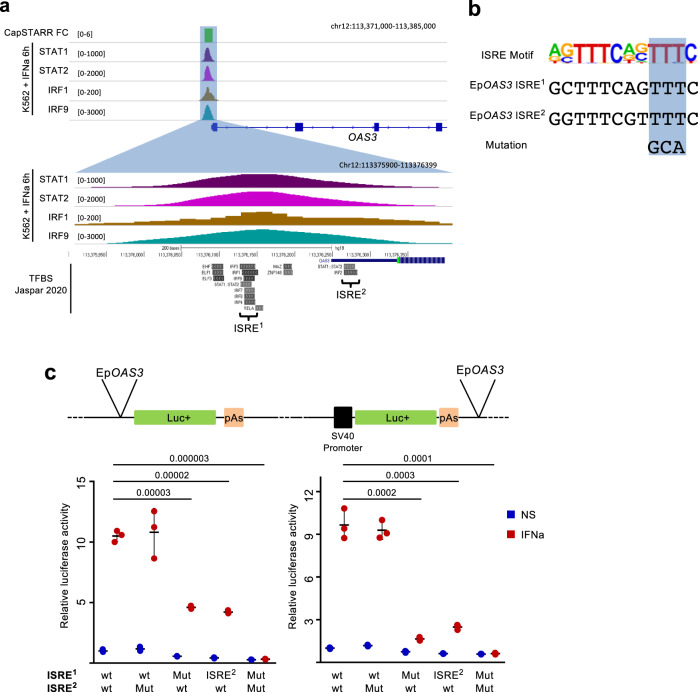
Luciferase assay of promoter and enhancer activity in wild-type and *OAS3* promoter mutants. **a** Genome browser tracks showing the fold-change of CapSTARR-seq activity and the ChIP-seq signal of the indicated TFs surrounding the *OAS3* Epromoter. The lower panel shows the location of significant TF-binding sites (Jaspar 2020) and the relative position of the two Interferon-Stimulated Response Element (ISRE) motifs in comparison with the ChIP-seq peaks. **b** Consensus sequence of the ISRE motif, as well as, the genomic sequence of the two identified ISREs in Ep*OAS3*, and the mutation made for the luciferase test is highlighted. **c** Luciferase assays to quantify the promoter (left) and the enhancer (right) activity of the wild-type (wt) *OAS3* Epromoter or with the indicated mutations (Mut) in non-stimulated (blue) or IFNa-stimulated (red) in K562 cells. Central values represent the median of the signal and the error bars represent the s.d. of three independent replicates. *P*-values were calculated by two-sided Student’s *t*-test.

A key question is whether enhancer and promoter activities are dictated by the same or distinct regulatory sequences. To start addressing this issue we analyzed the TF-binding sites IFNa-inducible *OAS3* Epromoter. The *OAS3* Epromoter harbors two ISRE-binding sites (hereafter ISRE^1^ and ISRE^2^), as well as a RELA-binding site (NFkb) in proximity to ISRE^1^ (Fig. 5a). Notably, ISRE^1^ corresponds to a canonical IRF9 motif and is closer to the edge of the STAT1/2 and IRF1/9-binding peaks (Fig. 5a, b). We set up a luciferase reporter assay to assess the contribution of each binding site to the enhancer and promoter activities. As expected, the *OAS3* Epromoter displayed IFNa-inducible promoter and enhancer activities in a luciferase assay (Fig. 5c). Mutation of the ISRE^1^ site, or replacement of ISRE^1^ by ISRE^2^, impaired enhancer activity by five-fold and promoter activity by two-fold, while mutation of the ISRE^2^ or the NFkb sites had no significant effect (Fig. 5c and Supplementary Fig. 8e), suggesting that enhancer activity of the *OAS3* Epromoter is more dependent on the presence of a “consensus” ISRE. The presence of the two ISRE-binding sites, however, was absolutely required for the IFNa-dependent activity. In conclusion, the enhancer and promoter activities of the *OAS3* Epromoter rely on the same regulatory motifs but display different sensitivities with respect to the similarity of the sequences to the consensus ISRE motif.

### Epromoters are involved in the regulation of an LPS-induced cluster in macrophages

**Fig. 6:**
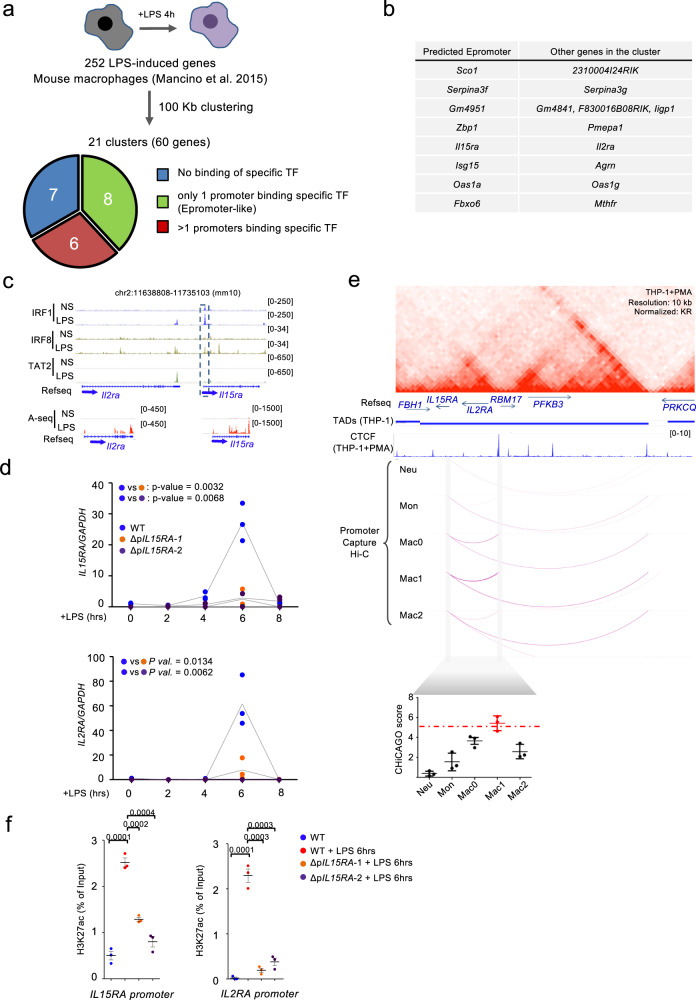
Epromoter-like clusters identification and validation of enhancer activity in LPS stimulated macrophages. **a** Schematic description of the clustering of the Lipopolysaccharide (LPS) induced genes in the mouse macrophages and pie-chart with the proportion of promoters that are bound by none (blue), 1 (green) or more than one (red) of the transcription factors (TFs) IRF1, IRF8 and STAT2, using data from Mancino et al.^29^. LPS induced genes in the mouse macrophages were clustered together if at least two of their promoters were located within less than 100 kilobases (kb). **b** Table showing the list of the 8 LPS-induced clusters with putative Epromoter elements as defined in **a**. **c** Genomic tracks centered on the mouse *Il15ra/Il2ra* cluster and showing the IRF1 (blue), IRF8 (bright olive) and STAT2 (green) ChIP-seq signal as well as the RNA-seq in unstimulated (NS) and after 4 h of LPS stimulation (LPS) of mouse macrophages. **d** qPCR analysis of gene expression of *IL15RA* and *IL2RA* genes in wild-type (blue) and Δp*IL15RA* mutants 1 (orange) and 2 (purple) of in vitro differentiated THP-1 macrophages in unstimulated and at different intervals of time of LPS stimulation. Values represent the relative expression levels as compared to the unstimulated conditions and normalized by the *GAPDH* housekeeping gene. Points indicate values of *n* = 3 independent experiments and the line in gray present the mean of the values. *P*-values were calculated using Two-Way ANOVA test. **e** Top panel: genomic tracks showing Hi-C triangular matrix (resolution at 10 kb, Knight-Ruiz (KR) normalized) and ChIP-seq CTCF signal in in vitro differentiated human THP-1 macrophages. Topological Associated Domains (TADs) from THP-1 cells^82^. Middle panel: DNA interactions detected by CHiCAGO at the *IL15RA* from Promoter Capture Hi-C from Javierre et al.^36^, in human primary neutrophils (Neu), monocytes (Mon), in vitro differentiated macrophages unstimulated (Mac0), stimulated with LPS (Mac1) or with IL-13 (Mac2). Bottom panel: scatter plot showing the mean +/− s.d. of the CHiCAGO score of the three interactions between *IL15RA* to the three bins surrounding the CTCF site downstream *IL2RA* in addition to a red line indicating the threshold. The data was taken from Phanstiel et al.^35^. **f** H3K27ac ChIP-qPCR on *IL15RA* and *IL2RA* promoters in the wild-type NS (in blue) and in the wild-type and Δp*IL15RA* mutants 1 and 2 of in vitro differentiated THP-1 macrophages upon 6 h of stimulation with LPS in red, orange and purple, respectively. Points indicate values of *n* = 3 independent experiments and the scatter plots shows the mean of values +/− s.d. *P*-values were calculated by two-sided Student’s *t*-test.

Based on the above results, we reasoned that within a cluster of induced genes in response to extracellular signaling, the promoter that preferentially recruits the key TFs can be predicted as having an Epromoter-like function. We used this prediction to explore the function of Epromoters in other inflammatory responses, namely the primary immune response of macrophages. For this purpose, we analyzed a dataset including stimulation of mouse primary macrophages by Lipopolysaccharides (LPS)^29^. The dataset consisted of RNA-seq as well as ChIP-seq for the IRF1, IRF8, and STAT2 TFs before and after LPS stimulation for 4 h. Briefly, we identified clusters of co-induced genes (for which the TSS are separated by less than 100 kb). Then, determined how many promoters from the cluster recruit a given TF (Fig. 6a; see Methods). Clusters harboring only one promoter binding at least one of the three aforementioned TFs, while the other promoters were devoid of binding of any of the TFs, were considered as potentially regulated by Epromoters (i.e., Epromoter-clusters). From 252 LPS-induced genes, we identified a total of 21 induced clusters (consisting of 60 genes) (Fig. 6a and Supplementary Data 6). Of these, 8 clusters were classified as Epromoter-like clusters (Fig. 6b). For instance, we identified the *ISG15-AGRN* cluster (Supplementary Fig. 9a), which was also validated as an Epromoter-dependent cluster in the IFNa response.

To further validate the approach, we selected the *Il15ra/Il2ra* cluster (Fig. 6c) coding for the receptors of the IL15 and IL2 cytokines, respectively. IL15RA (also known as CD215) plays a major role in the modulation of the pro-inflammatory response of LPS-stimulated macrophages^30^ and in supporting the homeostatic proliferation of CD8 + T lymphocytes^31^. IL2RA (also known as CD25) is mainly known for its role in T cell differentiation and activation^32^. Previous studies, however, have observed induction of *IL2RA* and responsiveness to IL2 in LPS-stimulated macrophages^33,34^. This suggests a role of IL2RA in the macrophage-mediated immune response. We observed that the IRF1 and IRF8 factors were bound to the *Il15ra* promoter upon LPS stimulation, but no binding was observed at the *Il2ra* promoter (Fig. 6c). We predicted that the *Il15ra* promoter might work as an Epromoter to coordinate the induction of both *Il15ra* and *Il2ra* genes after the LPS-stimulation of macrophages.

To experimentally validate our hypothesis, we used PMA-induced in vitro differentiated macrophage from the human THP-1 monocyte cell line, a classical model to study macrophage function (Supplementary Fig. 9b, c). We observed that both *IL15RA* and *IL2RA* genes were induced by LPS stimulation of in vitro differentiated THP-1 macrophages with similar kinetics and reached their maximum induction after 6 h (Fig. 6d), in line with the co-regulation observed in mouse macrophages. Next, we examined the 3D chromatin organization of the human IL15RA/IL2RA locus. CTCF binding and Hi-C data in macrophage-differentiated THP-1 cells^35^ revealed that both genes are in the same TAD within a regulatory subdomain flanked by CTCF (Fig. 6e). Moreover, analysis of Promoter Capture Hi-C interactions^36^ centered on the *IL15RA* promoter showed that the contact frequency (ChICAGO score) with the *IL2RA-*proximal CTCF site increase from monocyte to macrophage differentiation (M0) to reach the highest contact frequency in LPS stimulated macrophages (M1), but not in Il13-stimulated macrophages (M2) (Fig. 6e, bottom panels). Deletion of the *IL15RA* promoter in THP-1 cells (Supplementary Fig. 9d) resulted in significant impairment of both *IL15RA* and *IL2RA* induction after 6 h of LPS stimulation of in vitro differentiated THP-1 macrophages (Fig. 6d). Consistently, the gain of H3K27ac observed at both *IL15RA* and *IL2RA* promoters after 6 h of LPS stimulation of wild-type in vitro differentiated THP-1 macrophages was significantly reduced in *IL15RA* promoter deleted clones (Fig. 6f). Therefore, the *IL15RA* promoter function as a bona fide Epromoter to regulate both *IL15RA* and *IL2RA* induction in LPS stimulated macrophages as predicted by the binding profiles of involved TFs and the dynamic of 3D interactions. More generally, we show that it is possible to predict inducible Epromoter elements based on the binding profile of key TFs and that Epromoter-like regulatory elements play a role not only in type I interferon response, but also in other inflammatory responses, such as macrophage activation.

## Discussion

By systematically assessing gene expression and enhancer activity of coding gene promoters in response to type I interferon stimulation we found that a subset of IFNa response genes was associated with Epromoters. IFNa-induced Epromoters associated with an induced gene were found to preferentially recruit the key interferon response factors (STAT1/2, IRF1/9) and to be required for the efficient induction of neighbor genes within the same cluster. Functional studies in LPS-stimulated macrophages suggested that Epromoters play an important role in other inflammatory responses as well.

Previous studies suggested that Epromoters might be involved in stress response and, in particular, the interferon response^5,14,15^. In our previous study, we found that active Epromoters in HeLa cells, which have a constitutive interferon response, are significantly associated with stress and interferon response^5^. Indeed, inhibition of type I interferon in HeLa cells preferentially affects the activity of Epromoters as compared with distal enhancers^14,15^ (Supplementary Fig. 1). Moreover, distal genes interacting with Epromoters in both Hela and K562 cells are significantly enriched in the interferon response^5^. Induced Epromoters are associated with highly induced genes and correlate with a higher representation of bona fide interferon-response genes (i.e., conserved ISGs). IFNa-response Epromoters can be found in the proximity of other induced genes and ensure coordinated regulation of interferon response clusters. Notably, IFNa-induced genes with no Epromoters were significantly enriched for interferon-related GO terms arguing that they are also bona fide interferon response genes. We observed that IFNa-induced clusters were generally contained within a single TAD (Fig. 3e), consistent with their potential co-regulation. While unclustered genes are more likely to be regulated by typical enhancers, our results suggest that clustered genes are preferentially regulated by Epromoters. Overall, we suggest that distal regulation by interferon response Epromoters might play an important role in the coordinated response to IFNa-mediated signaling.

A general question about the Epromoter function is related to the mechanistic bases leading to their enhancer activity^2,37^. In a previous study, we showed that Epromoters bind a higher number of TFs and harbor a more complex combination of TF-binding sites as compared with classical promoters^5^, suggesting that one of the potential mechanisms mediating enhancer function might be the efficient recruitment of key TFs. Here, we made the striking observation that essential type I interferon-response factors (STAT1/2, IRF1/9) are preferentially recruited by induced Epromoters, which represents a minority (~8%) of the IFNa-induced promoters in K562 cells. In other words, the vast majority of promoters associated with IFNa-induced genes do not recruit the TFs that are essential for their activation. This is surprising given that it is commonly acknowledged that ISG promoters harbor the ISRE sites and that binding of the ISGF3 complex is required for the induction of ISGs^16^. Moreover, IFNa-induced activation of Epromoters was also associated with a preferential gain of activating histone modifications, H3K4me1, H3K4me3 and H3K27ac, suggesting that they represent the main IFNa-response promoters. Of note, we observed that several clusters of induced genes were associated with a constitutive Epromoter (Fig. 3H). However, constitutive Epromoters were not found to recruit the ISGF3 complex, thus the functional role of these Epromoters in the IFNa response will require further investigation.

Our study suggests that the majority of ISGs are regulated by distal regulatory elements, which could be either typical enhancer or Epromoter associated with another gene. Unbiased analysis of ISGF3 complex binding demonstrated that for a significant subset of IFNa-induced genes, the closest binding of ISGF3 was observed within a promoter of another gene. Indeed, we were able to identify several IFNa-response clusters that are associated with at least one Epromoter. Visual inspection of the epigenetic and TF-binding profiles at typical type I IFN response clusters, such as *IFIT* and *OAS*, suggests that no potential distal “intergenic” enhancers are found within the TADs containing these clusters (Supplementary Fig. 6). Experimental validation by CRISPR/Cas9 genome editing at three independent clusters demonstrated that the Epromoters were required for accurate induction of the ISGs within the same cluster.

Based on the binding of the ISGF3 complex, we suggest that ISGs within clusters are more likely to be regulated by Epromoters while ISGs outside clusters are more likely to be regulated by typical enhancers. Indeed, we found 21 clusters included at least one Epromoter as defined by the STARR-seq. However, the number of genes regulated by Epromoters might be underestimated by several reasons. On the one hand, we have used a stringent criterion of clustering based on a maximum of 100 kb between the TSS of induced genes. Indeed, analyses of TADs identify three additional clusters containing either induced or constitutive Epromoters (DHX58, TMEM140 and SIGLEC14; Supplemental Dataset 5) that were missed by the 100 kb clustering. Yet, it is plausible that clusters of induced genes might be found in a poorly interacting region before stimulation (e.g., clusters found as “outside” TADs in Fig. 3h) or that TAD borders might change upon stimulation. For instances, the OAS1/2/3 cluster was separated by to TADs in unstimulated K562 cells but were found to functionally interact after IFNa stimulation. On the other hand, the number of Epromoters might be underestimated by the STARR-seq approach. It is well accepted that STARR-seq as other similar reporter approaches have a low rate of sensibility^4^.

Besides the quantitative aspects about how many ISGs might regulated by Epromoters, our study points to a key role of Epromoters in the interferon response. First, induced genes associated with and induced Epromoter are more significantly associated with conserved ISGs, as compared to other induced genes. This included key interferon response genes such as *OAS1-3*, *IFIT1-5* and *ISG15*. Second, the analyses of distal and proximal enhancers in HeLa cells showed that the activity of Epromoters was significantly more affected than distal enhancers to inhibition of the IFN signal. Third, the binding of key IFN-response TFs and the density of associated binding sites is higher at induced Epromoters as compared to the promoters of other induced genes, but similar to the predicted distal enhancers. Fourth, the increase of histone modifications and gene expression associated with induced Epromoters are significantly higher than at the “induced gene only” set.

What can explain the favored recruitment of STAT-IRF complexes to the induced Epromoters? Although promoters of induced genes are generally enriched in ISRE, consistent with previous results^16^, the induced Epromoters were found to contain a higher number of ISRE-binding sites. This can lead to increased efficiency of TF recruitment and/or stabilization required for enhancer function. Features defining the enhancer versus promoter activity of regulatory elements are a fundamental question in the gene regulation field and a focus of extensive research^8,37–42^. Our study suggests that it is both the higher density and the better quality of key TF-binding sites that allow the efficient activation of the Epromoters and mediate its enhancer function. Indeed, mutation of one out of two ISRE in the *OAS3* Epromoter resulted in a more marked effect on its enhancer as compared to its promoter activity. Several studies have also suggested that the degree of enhancer or promoter activity is reflected by the levels and directionality of eRNA transcription^38–43^. We have previously shown that Epromoter activity is associated with the presence of unstable anti-sense transcripts, which are reminiscent of eRNA transcription, as found at typical enhancers^5^. It will be of interest to assess the contribution of eRNA in the function of IFNa-response Epromoters. Overall, our results show that for a subset of IFNa-regulated clusters, the Epromoter functions as a local hub efficiently recruiting the key TFs required for the coordinated regulation of the neighbor ISGs.

The use of high-throughput reporter assays provides the ability to test thousands of sequences in a certain nuclear environment, and pinpoint candidates with enhancer/promoter activity with or without any specific cellular stimulation. However, these activities do not necessarily reflect the actual function of any given sequence in their natural context, which might be influenced by several additional factors, including chromatin composition, histone modifications, DNA modifications, and the presence of other cis-regulatory elements^4^. As highlighted by the results obtained with the *OAS1-3* cluster, it is the preferential in vivo binding of the IFNa-response factors to one of the promoters of the co-regulated genes, within a given cluster, that better predict the enhancer activity in the endogenous locus. Indeed, while all three promoters of the *OAS1-3* cluster displayed enhancer activity when assessed by CapSTARR-seq, only the *OAS3* promoter, which efficiently recruits the STAT1/2 and IRF1/9 factors, was required for the proper induction of the other *OAS* genes. Importantly, analyses of non-deleted clones, as well as *OAS3* rescue experiments further support a direct role of *OAS3* Epromoter in the activation of the other two *OAS* genes. Based on the analyses of RNA-seq and ChIP-seq of STAT2, IRF1, and IRF8 in primary mouse macrophages stimulated by LPS, we identified several LPS-response clusters potentially regulated by Epromoters, including one also identified in K562 cells after IFNa stimulation. Experimental validation of one of these loci (*IL15RA*-*IL2RA*) demonstrated the predictive value of this approach and suggests that the involvement of inducible Epromoters might also apply to other types of inflammatory responses. This is also consistent with previous findings that promoters highly induced during the immune challenge of macrophages are characterized by enhancer-like features^38,43^.

Aside from our current evidence for the role of Epromoters in the interferon and immune response, it is likely that this type of regulatory element could be more generally involved in the rapid response of genes to cellular stress. Noteworthy, many of the early characterized enhancers are located close to, or overlapping with, the promoter region of inducible genes, such as metallothioneins, histones of early cleavage stages, viral immediate-early genes (from some papovaviruses, cytomegaloviruses, and retroviruses), heat-shock genes and the antiviral interferon genes^3,44^. A common characteristic of most of the aforementioned promoters is that they are associated with inducible genes that have to quickly respond to environmental stress. We hypothesize that, within clusters of co-regulated genes, enhancer-like promoters play an essential role in the coordination of rapid gene induction during the inflammatory response, and likely upon other cellular responses to intra- and extracellular stress signaling. This idea fits well with the notion of inducible transcription factories defining discrete membrane-less sub-nuclear compartments containing a high concentration of RNA polymerase and key TFs and where efficient transcription can be triggered^45,46^. A striking example is provided by NFkB-regulated genes in response to TNF alpha stimulation. Experimental removal of a gene from the NFkB-dependent multi-gene complex was shown to directly affect the transcription of its interacting genes, suggesting that co-association of co-regulated genes might contribute to a hierarchy of gene expression control^47^. The enhancer-like promoters could either facilitate the assembly or maintenance of the transcription factories by tightening promoter-promoter interactions or bringing specific transcriptional regulators required for the regulation of the neighbor genes. This would be particularly relevant in the case of rapid and coordinated regulation of gene expression in response to environmental or intrinsic cellular stimuli. In support of this hypothesis, we found that the *IL15RA* and *IL2RA* co-regulated genes are within the same interacting loop before macrophage stimulation, but the interaction is increased upon LPS stimulation, in agreement with previous results suggesting that a pre-establish loop between cis-regulatory elements can facilitate rapid gene induction^48,49^. Our work provides a framework for future studies aiming to address the contribution of enhancer-like promoters in the formation or stabilization of transcription factories in response to different signaling. In particular, the development of a bioinformatic pipeline based on the analyses of RNA-seq and ChIP-seq data in different stimulatory conditions might allow us to identify clusters of stimulus-regulated genes where only one promoter recruits the key TFs and potentially plays enhancer-like functions.

Our work has general implications for the understanding of genome organization and gene regulation in normal and pathological contexts. First, the coordinated regulation by Epromoters has physiological relevance for the co-regulation of inducible genes as a single regulatory element might ensure the synchronized gene expression and fine-tune the response to extracellular signaling. For instance, in the case of the interferon response, complexity can arise from differences in the endogenous levels of signaling pathway components and IFN properties^50^. These differences may be integrated by Epromoters to coordinate the variations in the nature and number of genes that are transcriptionally up and down-regulated and as a result, lead to distinct biological outcomes.

Secondly, as Epromoters potentially regulate several genes at the same time and have the ability to efficiently recruit essential TFs, mutations in these regulatory elements are expected to have a pleiotropic impact in disease, namely inflammatory diseases. Indeed, several examples point toward the relation between disease-associated variants and disrupted Epromoters^3^. Previous studies suggested that Single Nucleotide Polymorphisms (SNPs) affecting distal gene expression are significantly enriched within Epromoters^5,13,51–53^ and that human genetic variation within Epromoters influences distal gene expression^5,54^. Based on our current results, it is expected that Epromoters might have a role in the etiology of inflammatory diseases. Inflammation is viewed as the driving factor in many diseases, including atherosclerosis, cancer, autoimmunity, and infections, and it is a major contributor to age-related conditions^55^. Although IFN signatures are induced by environmental cues, their amplitudes, time courses, and patterns of gene expression are modulated by genetic factors. Allelic variants that regulate gene expression have been associated with complex multigenic autoimmune diseases as well as monogenic interferonopathy, characterized by a type I IFN signature^56^. We forecast that dissecting the consequences of the genetic contributions to the risk of developing a systemic autoimmune disease can be more complex than previously anticipated, given that a variant associated with an Epromoter can have a complex contribution to the interferon response by influencing the expression of different genes involved in the interferon signaling pathway. For instance, a recent study integrating 3D interactions and GWAS found an association between the interferon gene *IFNA2* and a genetic variant lying within the promoter of *CDKN2B* in coronary artery disease^52^. More generally, it is plausible that genetic variants within Epromoters might differentially impact enhancer versus promoter activity. For instance, two studies working on a promoter-overlapping SNP associated with prostate cancer demonstrated that the alternative variant increases the enhancer activity of the promoter leading to increased expression of two distal transcripts directly involved in cancer progression^57,58^.

Finally, our findings have significant implications for the understanding of the evolution of regulatory elements and interferon response loci. On the one hand, recent works suggested that repurposing of promoters and enhancers facilitated regulatory innovation and the origination of new genes during evolution^39,59–64^. On the other hand, gene expansion has been shown to significantly contribute to the evolution of the IFN system and suggested to confer a selective advantage to the host species^21^. It is possible that during duplication of ISG, such as in the case of IFIT or OAS genes, ancestral promoters’ elements have acquired enhancer functions to coordinate the interferon response of the newly appeared ISG within the cluster. Similarly, the proximity to an Epromoter-associated ISG locus might provide a rapid co-option for neighbor genes to acquire new functions in the interferon response. This is consistent with our results indicating that Epromoters are preferentially associated with conserved ISGs. In this context, it will be interesting to re-examine the dynamic evolution of the interferon responses across different species^21^.

Overall, we show that Epromoters work as a local hub for recruiting key TFs required for the activation of type I interferon response genes and are required for the accurate induction of interferon-regulated clusters. Our study suggests that Epromoters play an essential role in the coordination of rapid gene induction in the inflammatory response, and likely upon other cellular responses to intra- and extracellular signals, and to establish connections with other distal response genes.

## Methods

### Cell culture

Cell line K562 (CCL-243), a chronic myelogenous leukemia cell line, was obtained from the ATCC (American Type Culture Collection) and maintained in RPMI 1640 media (Thermo Fischer Scientific) supplemented with 10% FBS (Thermo Fischer Scientific) at 37 °C and 5% CO_2_. Cells were passaged every 3 days at 2 × 10^5^ cells/mL and routinely tested for mycoplasma contamination. Interferon-alpha (Sigma Aldrich, SRP4594) was used to induce the IFN type I response. In all, 1 × 10^6^ wild-type and mutant cells were induced using a concentration of 50 ng/mL of IFNα in 2 mL culture for 6 h. For each group, three independent stimulations were made.

THP-1, a human acute monocytic leukemia cell line, was obtained from DSMZ (ACC 16). Cells were grown in RPMI 1640 media (Thermo Fischer Scientific) supplemented with 10% FBS (Thermo Fischer Scientific) at 37 °C and 5% CO_2_. Cells were passaged every 3 days at 10^6^ cells/mL and routinely tested for mycoplasma contamination. To induce macrophage differentiation, THP-1 cells were firstly plated on 6-well plates (2 × 10^6^ cells/well), in media containing 10 ng/mL phorbol 12-myristate 13-acetate (PMA; Sigma-Aldrich, P1585) for 48 h. After 48 h of incubation, the PMA containing media was replaced with fresh media, and cells were incubated for an additional 24 h. THP-1 in vitro differentiated macrophages were then stimulated by replacing media with 2 mL of fresh media containing 100 ng/mL of LPS (Sigma-Aldrich, L9143) for 0, 2, 4, 6 and 8 h, upon which the extraction of RNA was performed. For each group, three independent stimulations were made.

### Human promoter CapSTARR-seq

The CapSTARR-seq promoter library used in this study has been generated previously^5^ and was based on the capture of the regions −200 to +50 bp relative to the TSS of 20,719 human protein-coding genes as well as 370 random genomic regions using a human genome library with an average size of 300 bp. We transfected 50 millions of K562 cells with 1.25 mg of the CapSTARR-seq promoter library using the Neon Transfection System (Thermo Fisher Scientific; pulse voltage 1450 V, pulse width 10, pulse number 3) and after 18 h we treated the cells with IFNa (50 ng/mL) for 6 h in duplicate. After 24 h, we processed the cells according to the Starr-seq protocol^5,19^. Briefly, transfected (cDNA) and non-transfected (input) libraries were single-end sequenced on an Illumina NextSeq 500 sequencer (Supplementary Data 4). FastQ files were trimmed using sickle with -q 20 option and mapped to the hg19 reference genome using Bowtie2 with default parameters. Sam files were converted using SamTools and bed files were generated with BedTools (v2.17.0) “BamToBed” command. Fragment reads were extended to 314 nt, corresponding to the average size of the captured fragments. Coverage of captured regions was computed using BedTools “coverage” command for both transfected and non-transfected libraries. The coverage was normalized by Fragments per kilobase per million reads mapped (FPKM). The ratio of the Starr-seq coverage over the input (fold-change) was computed for each sample. Promoter regions with enhancer activity were defined using the inflection point of the ranked fold-change as a threshold. Promoter regions with an FPKM < 1 in the input library were removed (421 promoters). Finally, we obtained the enhancer activity of 17,941 promoters regulating the transcription of 14,188 genes (Supplementary Data 1). IFNa-induced Epromoters were defined as those Epromoters with an average ratio between IFNa-stimulated and non-stimulated conditions greater than two and considering that at least one replicate was active. Results are summarized in Supplementary Data 1 and 2.

### RNA-seq

Poly(A) RNA was isolated from three replicates of K562 cells non-stimulated or stimulated with IFNa for 6 h and used for RNA-seq library preparation, following the TruSeq RNA Library Prep Kit v2 (Illumina). Libraries were paired-end sequenced on an Illumina NextSeq 500 sequencer (Supplementary Data 4). Reads were aligned using STAR aligner (v2.4.2a) with arguments “outFilterMismatchNoverLmax” and “outFilterMultimapNmax” set to 0.08 and 1, respectively. Differential gene expression was performed using DESeq2 (v1.6.3) Bioconductor package^65^ implemented in the R statistical environment, and transcripts associated with promoters analyzed by CapSTARR-seq were retrieved. Genes with changes in expression with a log_2_ fold-change >1 and adjusted *P*-value <0.001 were considered as significant Supplementary Data 1 and 2). To create bigwig files reads from Watson and Crick strands were selected using SAMtools (v0.1.9) and provided to the bam2wig.py script from the RSeQC program suite (v2.6.4).

### ChIP and ChIP-seq

Cells were cross-linked with 1 % formaldehyde at room temperature for 10 mins followed by quenching with Glycine at 250 mM. Chromatin was sonicated using Bioruptor® Pico from 10 to 20 cycles (30 s ON, 30 s OFF) at 4 °C. The ChIP for the IRF9 TF was performed with 5 × 10^6^ K562 cells stimulated with IFNa for 6 h and 5 μL of IRF9 Rabbit mAB (Cell signal, D2T8M) in 300 μL of ChIP dilution buffer. ChIP for the histone modifications was performed with 5 × 10^5^ of K562 cells non-stimulated and stimulated with IFNa for 6 h, or 5 × 10^5^ of in vitro differentiated THP-1 cells non-stimulated and stimulated with LPS for 6 h, and 1 μg of mAB against H3K27ac, H3K4me3 or H3K4me1 (Diagenode, C15210016, C15410003 and C15410194, respectively) in 300 μL of ChIP dilution buffer. Chromatin was incubated with the antibody for overnight at 4 °C followed by the addition of 20 μL of Dynabeads protein G (Life technologies, 10004D). Bound immune complexes was washed three times and finally incubated in ChIP elution buffer (1% SDS, 0.1 M NaHCO3) and 2 μL of proteinase K (20 mg/mL, Life technologies 25530049) at 65 °C for overnight. DNA was then purified using QIAquick PCR Purification Kit (Quiagen, 28104). qPCR analyses were performed using the SYBR green Master Mix (Thermo Fisher Scientific) on the QuantStudio 6 instrument (Thermo Fisher Scientific). The relative expression was analyzed using the relative standard curve method and values were assessed as percentage of the input. Primers used are listed in Supplementary Data 7. ChIP-seq libraries were generated with the MicroPlex Library Preparation Kit (Diagenode), according to the manufacturer’s instructions. The libraries were sequenced in paired-end 50/30nt mode using the NextSeq® 500/550 (Illumina), according to the manufacturer’s instructions (Supplementary Data 4). Reads were trimmed with Sickle v1.33 and aligned to the hg19 genome assembly using Bowtie v2.3.4.3 and default parameters. ChIP-Seq coverage tracks (bigwig) were generated with deepTools^66^ v3.2.1 using -normalize RPKM. Peaks for ChIP-Seq were called using Macs2. ChIP-seq data for the IRF1, STAT1, and STAT2 TFs in K562 cells stimulated with IFNa were obtained from the ENCODE Consortium (Supplementary Data 4). Median average profiles were generated using the SeqPlots tool^67^ for the 2 Kb and 10 Kb regions centered at the TSS of the selected genes for the TFs (IRF9, IRF1, STAT1, and STAT2) and the histone modifications (H3K27ac and H3K4me3), respectively. ChIP-seq profiles were visualized using the IGV genome browser^68^.

### Functional enrichment

The GO enrichment for biological functions was performed using the webtool GREAT^69^ using the hg19 genome and default settings. The background regions used were all the captured promoters. Only the top 10 most significant terms are shown. The full list is provided in Supplementary Data 3.

### CRISPR/Cas9 genome editing

For the deletion of *OAS3*, *IFIT3*-P1, *OAS1*, and *OAS2* Epromoters in K562 cells we used the webtool CRISPRdirect^70^ to design two guide RNAs flanking the Epromoter region and clone them into the gRNA vector (Addgene #41824)^71^. Two primers were designed flanking the target region that allows us to identify the wild-type and the mutant alleles. For the deletion of Ep*OAS3*, Ep*ISG15*, and Ep*IFIT3* the transfection was made with 1 × 10^6^ K562 cells mixed with 2 μg of hCas9 vector (Addgene #41815) and 1 μg of each gRNA vector using the Neon Transfection System (Thermo Fisher Scientific; pulse voltage 1450 V, pulse width 10, pulse number 3) and cultured in 5 mL. For the deletion of Ep*OAS2* and Ep*OAS1* 1 × 10^6^ K562 cells were transfected with 0.5 µg of each gRNA and 1 µg of Cas9 (Addgene, #41815) in 20 µL transfection solution using the 4D-Nucleofactor X Unit (Lonza), P1 Primary Cell 4D-Nucleofactor X kit S, program FF-120. For the deletion of *ISG15*, and *IFIT3*-P2 Epromoter in K562 cells and for the *IL15RA* promoter in THP-1 cells, gRNAs were designed using CrispRGold^72^. We used the Alt-R CRISPR-Cas9 System from IDT (Integrated DNA Technology) where Ribonucleoprotein (RNP) complexes were assembled in vitro and then transfected by electroporation to the cells, accordingly to the manufactured instructions. Three days after transfection, cells were cultured in 3 × 96-well plates at limit dilution (0.5 cells/100 μL/well). After 2–4 weeks the clones were screened for homologous deletion using the kit Phire Tissue Direct PCR Master Mix (Thermo Fisher Scientific, F170L). Clones with homologous deletion were those showing a mutant band of the expected size and no wild-type band. For the CRISPR controls of the deletion of the *OAS3* Epromoter, we transfected 1 × 10^6^ K562 *wild-type* cells with 1 µg of each of the gRNA used for the *OAS3* Epromoter deletion. Sanger sequencing identified one clone with an inert mutation (single-base insertion) in the first intron of the *OAS3* gene. Primers and gRNAs are listed in Supplementary Data 7.

### *OAS3* gene expression recovery

To recover the expression of the *OAS3* gene, we transfected 1 × 10^6^ K562 Δ*OAS3* clones 1 and 2 with the human ORF clone of *OAS3* (ORIGENE, ref. RC222722) using the Neon Transfection System (Thermo Fisher Scientific; pulse voltage 1450 V, pulse width 10, pulse number 3). We used the expression plasmid of GFP (addgene, #46956) as a transfection negative control. After 2 h, wild-type and transfected cells were induced using a concentration of 50 ng/mL of IFNα in 2 mL culture for 6 h. For each group, three independent stimulations were made.

### Gene expression analysis

RNA was extracted using the RNeasy Plus Mini Kit (Qiagen) according to the manufacturer’s instructions with DNase treatment. One microgram of total RNA was reverse transcribed using the SuperScript II (Thermo Fisher Scientific). qPCR reactions were made using the SYBR green Master Mix (Thermo Fisher Scientific) on the QuantStudio 6 instrument (Thermo Fisher Scientific). 1:10 dilution of cDNA was used for the qPCR. The relative expression was analyzed using the relative standard curve method and the *GAPDH* gene was used for normalizing samples. For each group, three independent RNA and cDNA preparations were made. For the analyses of *Il15RA* and *IL2RA* genes in the THP-1 cell line, reverse transcription of 2,5 ug of RNA was done using Master Mix SuperScript™ VILO™ (Thermo Fisher Scientific, 11755250). 1:50 dilution of cDNA was used for the qPCR. qPCR reactions were made using the PowerUp SYBR Green Master Mix (Thermo Fisher Scientific, A25742) and the measurement was made using the Applied Biosystems QuantStudio 6 Flex Real-Time PCR System. The relative expression was calculated using the relative standard curve method from the mean of two technical replicates values according to the *GAPDH* gene expression. The mean value and standard deviations were calculated between the relative expression values of the three biological replicates, and values were afterward normalized by the value of the unstimulated WT. Primers used are listed in Supplementary Data 7.

### Luciferase reporter assay

The promoter of human *OAS3* (500 bp, Chr12:113375900–113376399) was cloned into the pGL3-Basic vector (Promega, E1751) upstream the luciferase gene at the BglII and HindIII restriction sites (p*OAS3*-luc), and into the pGL3-Promoter vector (Promega, E176; containing the SV40 promoter) downstream the luciferase gene at the BamHI and SalI restriction sites (pSV40-luc-p*OAS3*). Site-specific mutagenesis of the pOAS3 was done using the Q5 site-directed Mutagenesis Kit (NEB, E0554S) using a set of primers listed in Supplementary Data 7. For cell transfection, 1 × 10^6^ K562 cells were mixed with 1 μg of each construct and 200 ng of Renilla vector using the Neon Transfection System (Thermo Fisher Scientific; pulse voltage 1,450 V, pulse width 10, pulse number 3) and cultured in 2 mL in 12-well plates. After 18 h, half of the cell population was treated with 100 ng of IFNα (Sigma Aldrich, 50 ng/mL) for 6 h. Data were normalized to Renilla values and represented as the fold-change of relative light units over the wild-type pOAS3-luc or pSV40-luc-pOAS3 vector from non-stimulated K652 cells. Experiments were performed in triplicate.

### Genomic analyses of interferon-dependent enhancers in Hela cells

Interferon-dependent and independent enhancers in Hela-S3 cells were retrieved from the Muerdter et al. study^15^. The dataset comprises a genome-wide STARR-seq enhancer screening in HeLa-S3 cells treated or not with a combination of two inhibitors of type I interferon response. The annotation of enhancer regions with respect to the closest RefSeq TSS was performed using HOMER’s annotatePeaks.pl tool^73^. Based on the distance of active enhancers from the TSS, the genomic density distribution of active enhancers in inhibitor-treated and untreated conditions was calculated. Kolmogorov–Smirnov (KS) test was performed to compare the genomic distribution of active enhancers in the two conditions. Based on the distance to the nearest TSS, the enhancer regions were further divided into two categories: (I) TSS-proximal (≤1 kb from the closest TSS) and (ii) TSS-distal (>1 kb from the closest TSS) enhancer regions. Based on the distance of active enhancers from the TSS, the genomic density distribution of active enhancers in inhibitor-treated and non-treated conditions was calculated. Kolmogorov–Smirnov (KS) test was performed to compare the distribution of active enhancers in the different conditions.

### Motif enrichment analyses

Known motif enrichment and de novo motif discovery analyses for TFs-binding sites were performed using the findMotifsGenome.pl program in HOMER suit^73^ using the default motif lengths and 200 nt long sequences centered on each enhancer region as input. The ten enriched known motifs with the lowest adjusted *P*-value from each condition and dataset were extracted. For Muerdter et al. dataset, all proximal and distal enhancers for each condition were used as a background. A heatmap was plotted using the -log_10_ (adjusted *P*-value). Lower *P*-value corresponds to higher enrichment in dark color. For motif enrichment in K562 promoters, all promoters analyzed by CapSTARR-seq were used as a background. The top de novo motif with the lowest *P*-value from each promoter category was demarcated.

### ISRE binding across the promoter regions

The identified promoter regions were analyzed with respect to the enrichment of the interferon-stimulated response element (ISRE) and TFs individually. The binding site’s information of TFs, IRF family (IRF1–9), and STAT1/2 of the human genome (hg19) were extracted from the latest version of the JASPAR database^74^. The individual TFs-binding regions were generated by bedtools merge (at least 1 base pair overlap), whereas the general ISRE was generated using the bedtools merge in all the IRF family of TFs and STAT1/STAT2. The TF-binding occurrences in the different categories of promoters were computed by counting intersects (bedtool intersect) of individual TFs-binding regions and ISRE across corresponding promoter regions.

### Positional distribution of IFNa-specific TFs-binding sites

Overlapping binding sites of STAT1, STAT2, and IRF9 in IFNa-stimulated K562 cells were defined using the ‘intersect’ parameter from BEDTools v2.28.0^75^, based on ChIP-seq data. For each induced gene, we identified the closest merged peak. The location of the closest merged peak was categorized into three groups: (i) the same promoter, located within ±1 kb from the TSS of the induced gene; (ii) intergenic, located at >1 kb from the TSS of any RefSeq gene and; (iii) another promoter, located ±1 kb from the TSS of any other RefSeq gene. Statistical significance of the positional distribution was assessed using the OLOGRAM tool^76^ from the Pygtftk package^77^.

### Proximity of IFNa-induced genes in the human genome

To find the distribution of induced genes in the genome, the same number of IFNa-stimulated genes was extracted randomly from the human genome (hg19). The randomly selected genes were further clustered based on distance (≤100 kb) between the TSS of two induced genes. Further, the distance density distributions of observed and randomly selected genes were compared using Kolmogorov–Smirnov (KS) test.

### Identification of IFNa-dependent clusters

The TSS coordinates of IFNa-induced genes or associated with induced Epromoters in K562 cells (as defined in Supplementary Data 2) were extracted with reference to the RefSeq annotations. The distance between each pair of TSSs was computed using a custom Python script. A cluster of IFNa-induced loci was defined if at least two TSS of induced genes or Epromoters were found to be closer than 100 kb. The clusters were then classified in function of the type of genes they contained: “induced gene only”, “induced gene & Epromoter”, “induced Epromoter only”, or whether they contained a constitutive Epromoter (Supplementary Data 1). To assess whether the clustered genes were located within the same TAD, we used the coordinates of TAD borders identified in K562 cells from highly resolutive Hi-C data^78^. Overlapping domains were merged into one single TAD domain as described in^79^. The detailed clusters are summarized in Supplementary Data 5.

### Identification of LPS-dependent clusters in primary macrophages

LPS-induced genes (log_2_(FC) > 1; *P*-val. <0.01) and binding sites of interferon-associated TFs (IRF1, IRF8, STAT1) were extracted from the Mancino et al. study^29^. The dataset comprises gene expression and TF-binding sites (mm9) in mouse primary macrophages before and after LPS stimulation for 4 h. The TSS coordinates of LPS-induced genes were extracted with reference to the RefSeq annotations. LPS-induced clusters were identified based on distance (≤100 Kb) as described for the IFNa-response clusters. The promoters binding with TF(s) were considered only if the TF-binding site(s) overlapped at least 1 bp with the promoter region (±1 Kb from the TSS). We then determined the number of promoters per cluster that bind a given TF. The detailed description of the LPS-induces clusters is described in Supplementary Data 6.

### 3D chromatin organization of the *IL15RA/IL2RA* locus

CTCF ChIP-seq and Hi-C data in THP-1 + PMA cells from the data of Phanstiel et al. study^35^ were visualized using the New WashU Epigenome Browser (epigenomegateway.wustl.edu)^80^. TADs called from Hi-C experiments (HindIII) THP-1 cells were taken from^81^. DNA interactions centered on the *IL15RA* promoter with the associated CHiCAGO score were taken from published Promoter Capture Hi-C^36^ and visualized using the New WashU Epigenome Browser.

### Statistical analysis

GraphPad 7.0 was used for the statistical analysis of luciferase, ChIP-qPCR gene expression assays. For ChIP-qPCR and luciferase essays, two-sided Student’s *t*-test was performed. Statistical analysis of gene expression assay during different time of stimulation was done using two-way ANOVA test comparing the WT samples to each mutant. *P*-values are mentioned on the figure when it is significant.

### Reporting summary

Further information on research design is available in the Nature Research Reporting Summary linked to this article.

## Supplementary information

Supplementary Information

Description of additional Supplementary File

Supplementary Dataset 1

Supplementary Dataset 2

Supplementary Dataset 3

Supplementary Dataset 4

Supplementary Dataset 5

Supplementary Dataset 6

Supplementary Dataset 7

Reporting Summary

## Data Availability

The data that support this study are available from the corresponding author upon reasonable request. The RNA-Seq, CapSTARR-seq and ChIP-Seq data generated in this study have been submitted to the Gene Expression Omnibus (GEO) under the accession code GSE159462. Public datasets can be found under accession codes: ENCSR000FAU, ENCSR000FBC, ENCSR000EGL, ENCSR669GJD, GSE183296, GSE63525, GSE56123, GSE96800, GSE89663. All generated and publicly available datasets are listed in the Supplementary Data 4. Public databases used in this study are: Jaspar 2020 [http://genome.ucsc.edu/cgi-bin/hgTrackUi?hgsid=1169499883_qhnKn3jHG401qgfVk2LXUvfvZ2rU&db=hg38&c=chr19&g=hub_186875_JASPAR2020_TFBS_hg^38^]; Interferome database [http://www.interferome.org/interferome/home.jspx]. Source data are provided with this paper.

## Source data

Source data

## Code availability

All custom scripts used in this study are available at GitHub (https://github.com/Spicuglia-Lab/IFN-Data-Analysis) and archived at 10.5281/zenodo.5507612^83^.
