# Supplementary material for: Epromoters function as a hub to recruit key transcription factors required for the inflammatory response
Source: Nat Commun. 2021 Nov 18;12:6660. doi: 10.1038/s41467-021-26861-0 (PMC8602369; doi:10.1038/s41467-021-26861-0)
Supplement: Supplementary file 1 — Supplementary Information [file 41467_2021_26861_MOESM1_ESM.pdf]

Epromoters function as a hub to recruit key transcription factors required for the inflammatory response

David Santiago-Algarra, Charbel Souaid, Himanshu Singh, Lan T.M. Dao, Saadat Hussain, Alejandra Medina-Rivera, Lucia Ramirez-Navarro, Jaime A Castro-Mondragon, Nori Sadouni, Guillaume Charbonnier and Salvatore Spicuglia

Supplementary Fig. 1 Distribution and transcription factor motif enrichment of active enhancers in HeLa cells.

Supplementary Fig. 2 Interferon response in K562 cells.

Supplementary Fig. 3 Violin plot of the distribution of the binding sites of IRF1-9, and STAT1/STAT2 transcription factors per promoter.

Supplementary Fig. 4 Epigenomic characteristics of the closest ISGF3 binding relative to the "induced gene only" set.

Supplementary Fig. 5 Distance between induced genes and Epromoters and genomic visualization of the IFNa-induced cluster *APOBEC3.*

Supplementary Fig. 6 Genomic visualization of TADs harboring IFNa-induced clusters.

Supplementary Fig. 7 Genomic visualization and kinetic analysis of the IFNa induced clusters IFIT.

Supplementary Fig. 8 Gene expression of *OAS3* in the different mutants of the *OAS* cluster.

Supplementary Fig. 9 Macrophage stimulation by LPS.

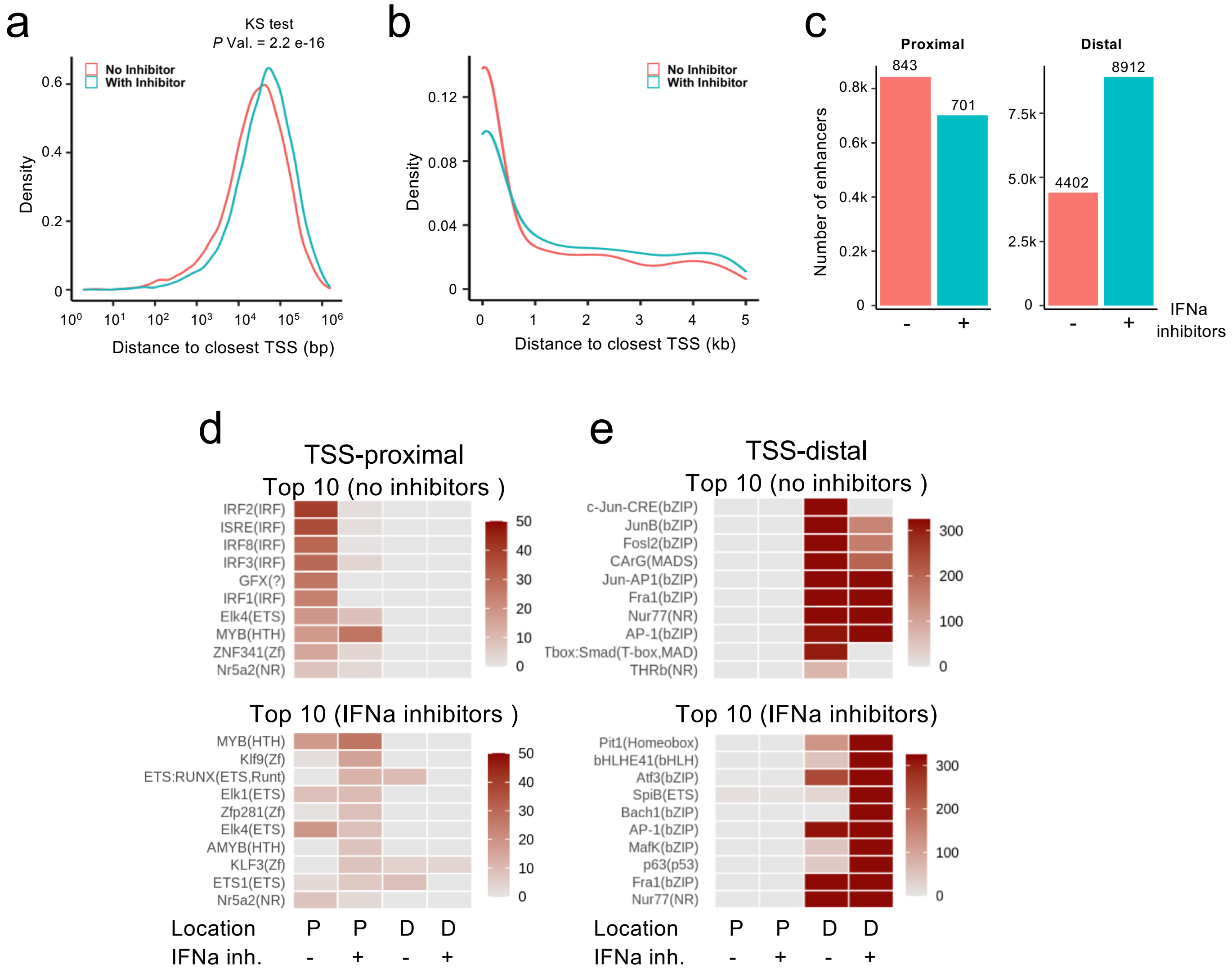

Supplementary Fig. 1 Distribution and transcription factor motif enrichment of active enhancers in HeLa cells. a-b Distribution of active enhancers in HeLa-S3 cells in function of the distance to the closest TSS with (blue) or without IFN response inhibitor (red), based on whole-genome STARR-seq data (Muerdter et al. 2018). Data plotted is the density of all active enhancers and the distance to the closest TSS (**a**; $\log_{10}$ scale) and the density of active enhancers located less than 5 kb to the closest TSS (**b**). Two-sided Kolmogorov–Smirnov (KS) test was used to compare the distribution of active enhancers in the presence or absence of interferon inhibitors. **c** Number of proximal (≤1 kb from the TSS) and distal (>1 kb from any TSS) enhancers identified in the presence or absence of IFNa inhibitors. **d-e** Motif enrichment analysis of TSS-proximal (**d**; ≤1 kb from the TSS) and distal (**e**; >1 kb from any TSS) enhancers in HeLa-S3 cells in the presence (bottom) or absence (top) of interferon inhibitors, using the HOMER tool. The top 10 enriched motifs for each condition were retrieved and the enrichment score (inverted Log_{10} of the adjusted P value) computed for the other conditions. The enrichment score was then plotted as a heatmap. When the motif was not found to be significant, the enrichment score was set to 0.

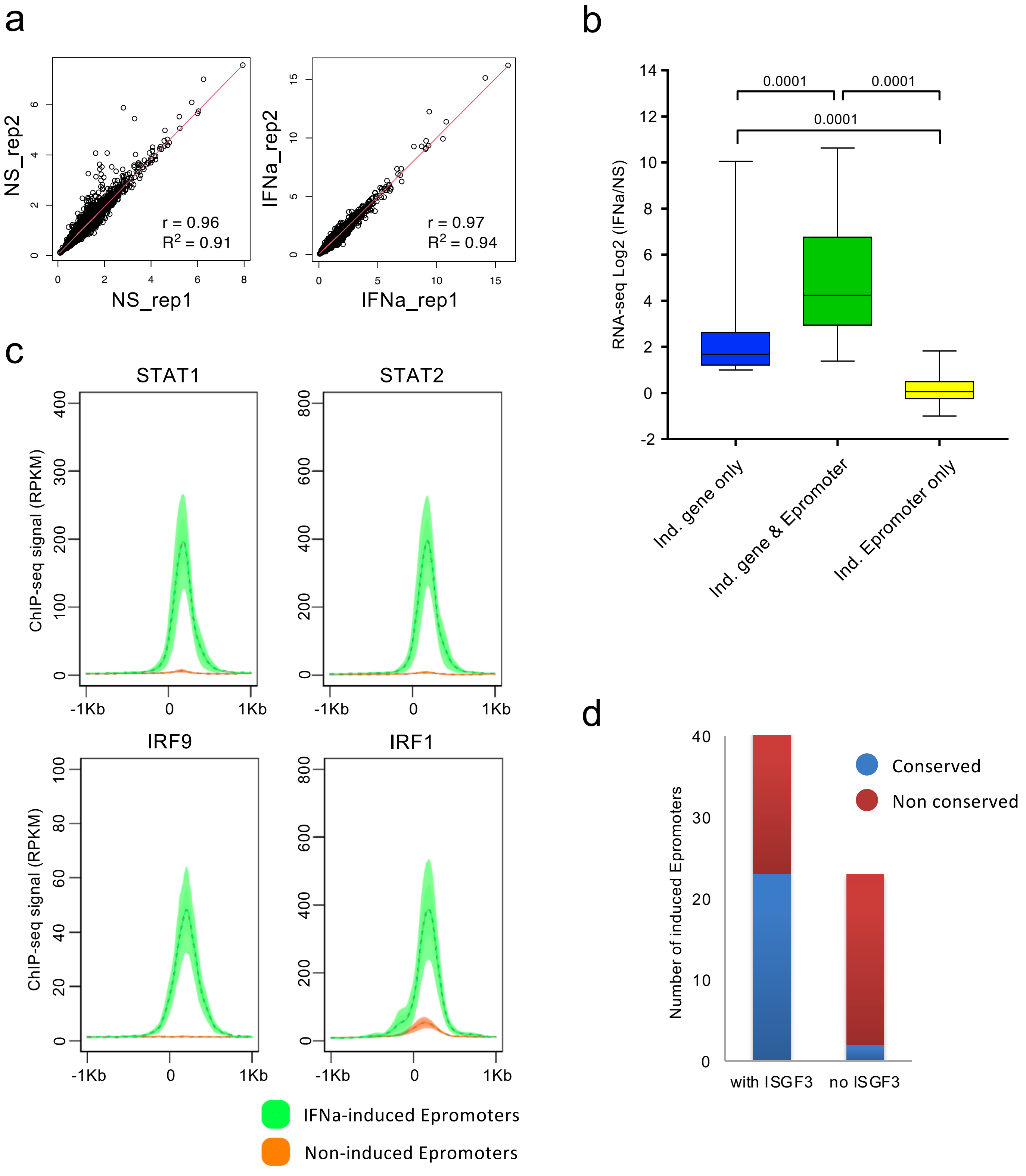

Supplementary Fig. 2 Interferon response in K562 cells. a Distribution of CapSTARR-seq signal between replicates. The loess regression curve as well as Pearson's r coefficients are displayed. **b** Boxplot of RNA-seq signal of the different IFNa-induced sets. Data plotted is the log2 of the FPKM ratio between K562+IFNa and K562-NS in three biological replicates. Central values represent the median of the signal, the interquartile range (IQR) corresponds to the 75th to 25th percentile, and whiskers extend to the maximum and minimum values excluding outliers. Two-sided Kruskal-Wallis test was performed and *P* values between the three groups are annotated. **c** Average profiles of ChIP-seq signals for the transcription factors STAT1, STAT2, IRF9, and IRF1 in K562 cells stimulated or not with IFNa for 6h, for the constitutive and IFNa-induced Epromoters as defined in Supplementary Table 1. The solid line represents the mean of the signal while the colored area represents the 95% CI. **d** Number of induced Epromoters bound by the ISGF3 complex. The proportion of Epromoters associated with conserved ISGs is also indicated.

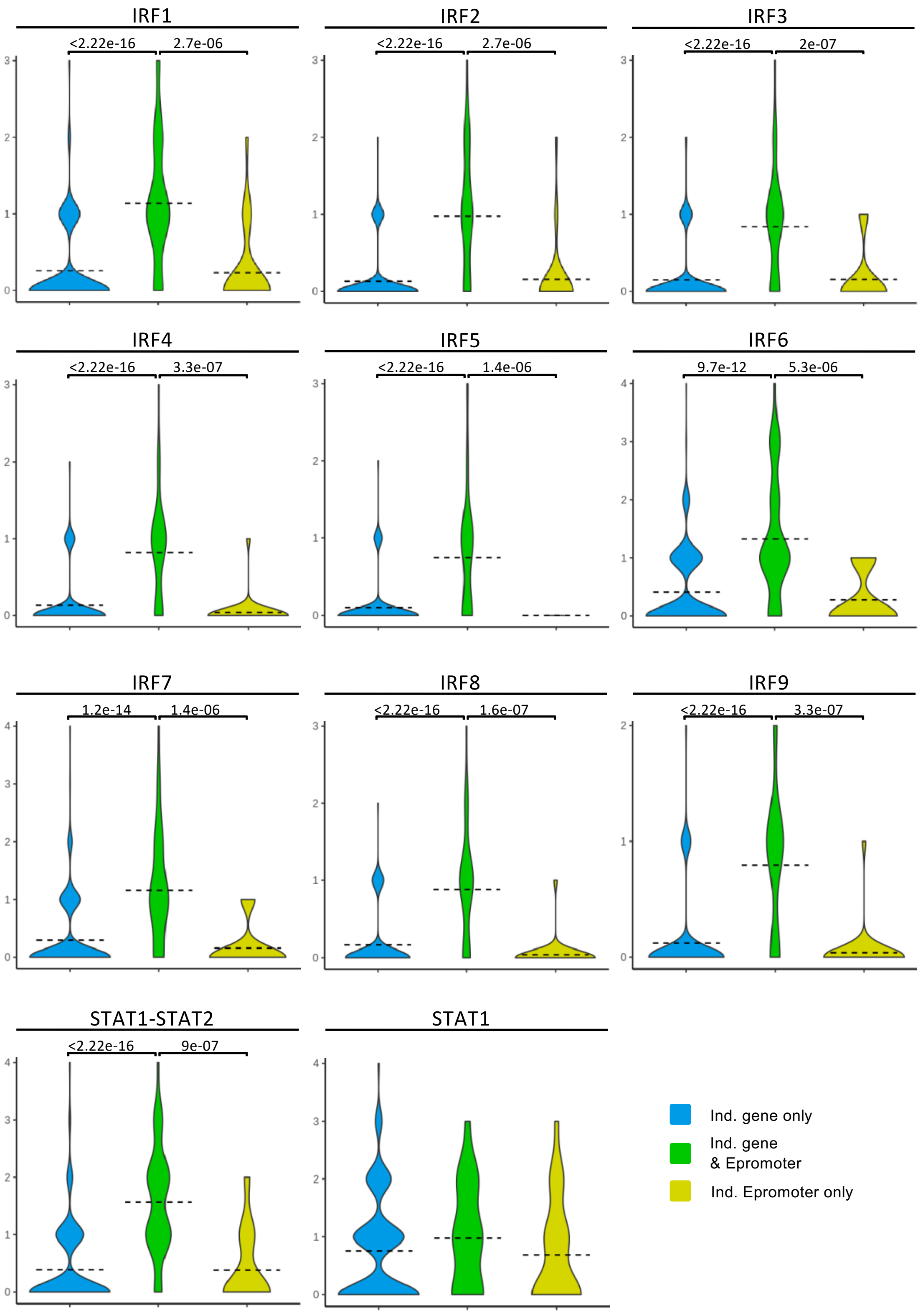

Supplementary Fig. 3 Violin plot of the distribution of the binding sites of IRF1-9, and STAT1/STAT2 transcription factors per promoter. Data shown is the number of the TFs binding sites in the groups defined in Supplementary Table 2. Dashed lined, mean. Only significant *P* values in the Kruskal-Wallis test are shown.

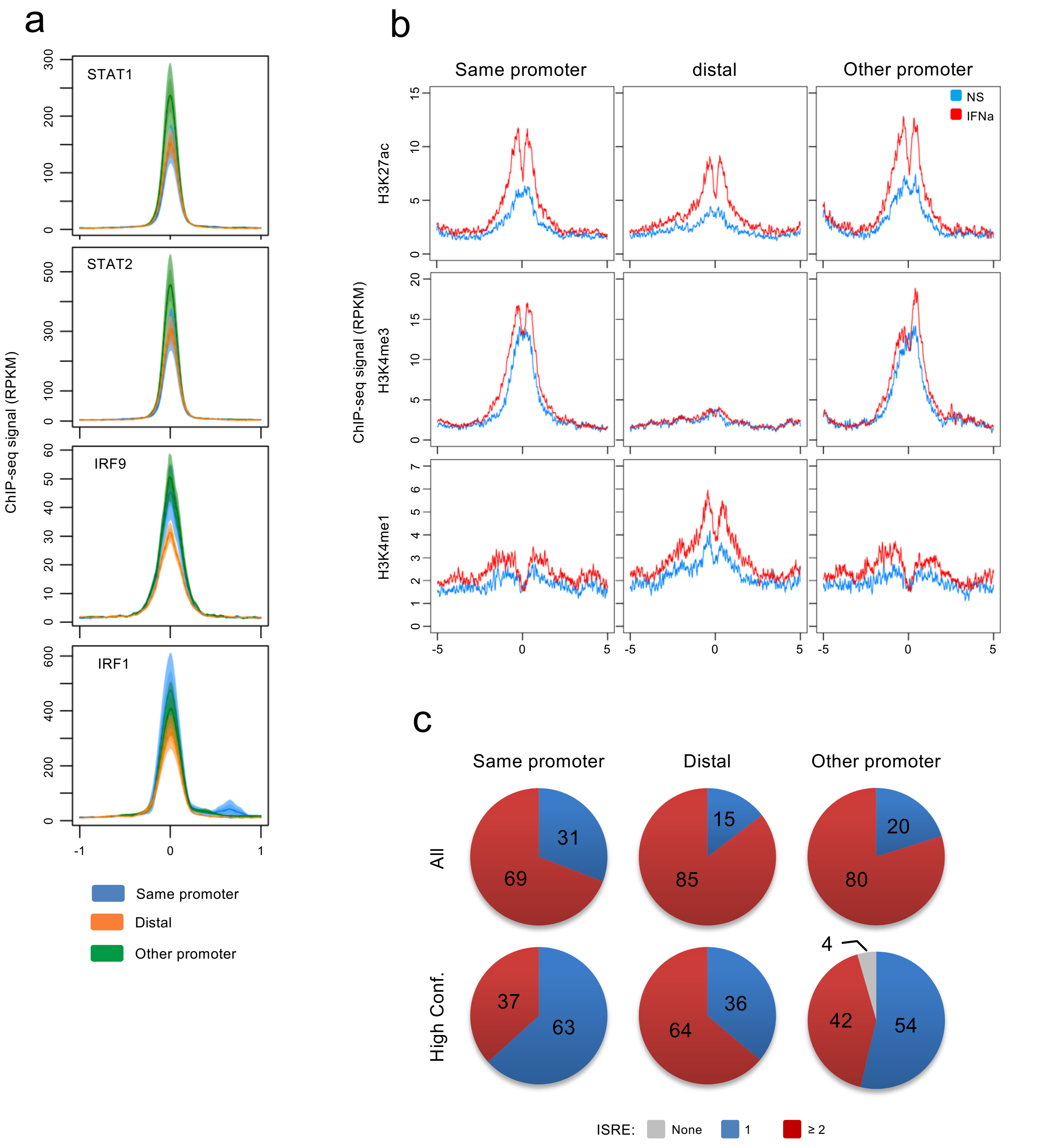

Supplementary Fig. 4 Epigenomic characteristics of the closest ISGF3 binding relative to the "induced gene only" set. a Average profiles of ChIP-seq signals for the transcription factors STAT1, STAT2, IRF9, and IRF1 in the closest ISGF3 binding peaks at the same promoter (blue; ≤1 Kb from the TSS of "induced gene only"; another promoter (green; ≤1 Kb from the TSS of other genes), or intergenic region (orange; >1 Kb from any TSS of a coding gene) in K562 cells stimulated with IFNa for 6h. The solid line represents the mean of the signal while the coloured area represents the 95% confidence interval. **b** Average profile of ChIP-seq signals for the histone modification H3K27ac, H3K4me1 and H3K4me3 in K562 cells non-stimulated (blue) and stimulated with IFNa for 6h (red). The solid line represents the mean of the signal. **c** Pie-charts showing the percentage of promoters from the indicated data sets that contain either none (grey), one (blue) or two or more (red) ISRE binding sites (merged sites for IRF1-9 and STA1-2 motifs) based on the Jaspar 2020 annotation74 by default (All) or with a *P* value < 1e-4 (High confidence).

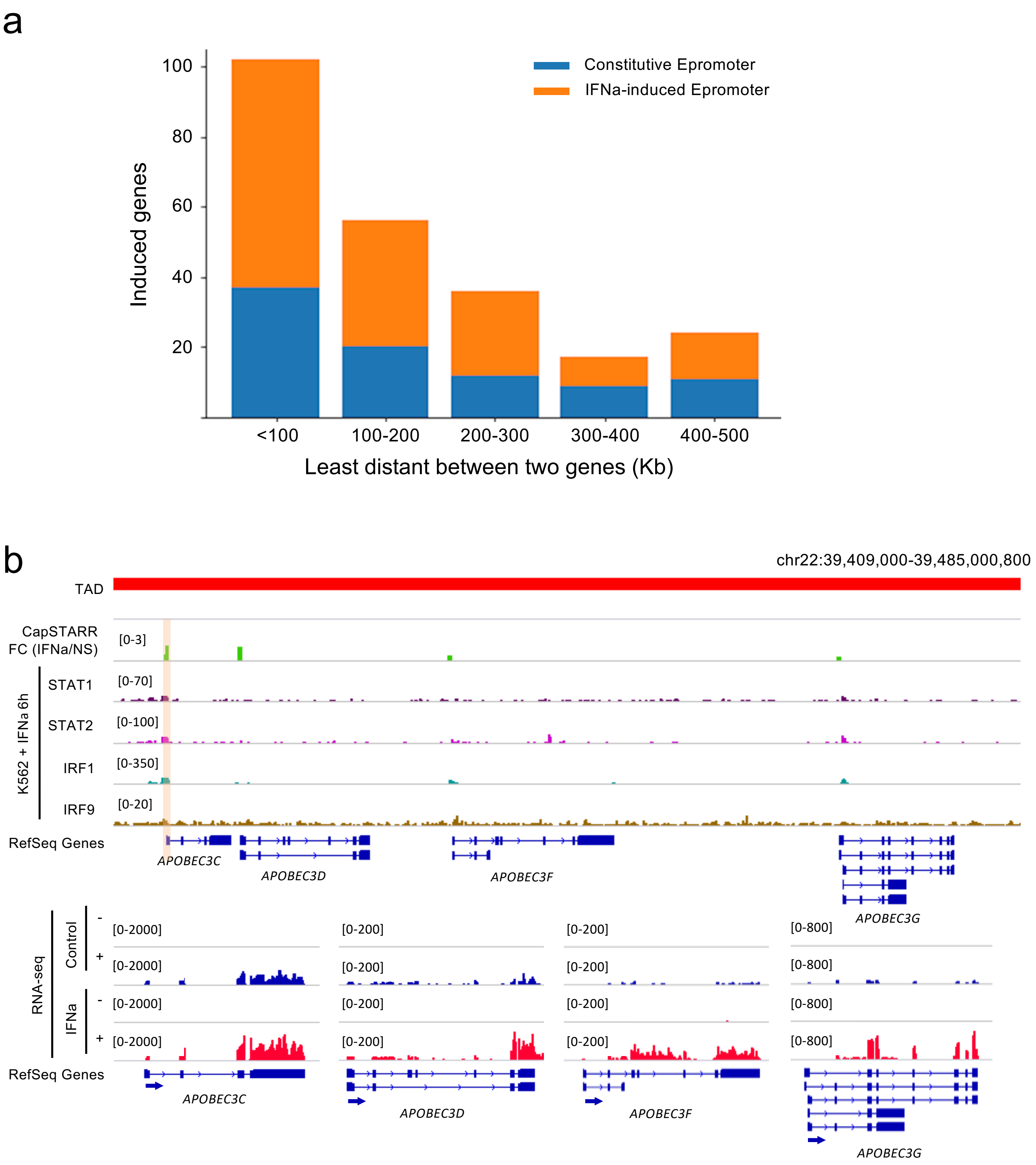

Supplementary Fig. 5 Distance between induced genes and Epromoters and genomic visualization of the IFNa-induced cluster *APOBEC3*. *a* Distribution of the distance between IFNa-induced genes and constitutive Epromoters and IFNa-induced Epromoters in K562. **b** Top panels show the TAD, CapSTARR signal fold change (IFNa over non-stimulated) and the ChIP-seq signal for the indicated transcription factors after IFNa induction with the Epromoter of the *APOBEC3C* highlighted. Bottom panels show the RNA-seq signals of the induced genes. Positive and negative signs indicate the RNA-seq signal on the sense and anti-sense strands, respectively.

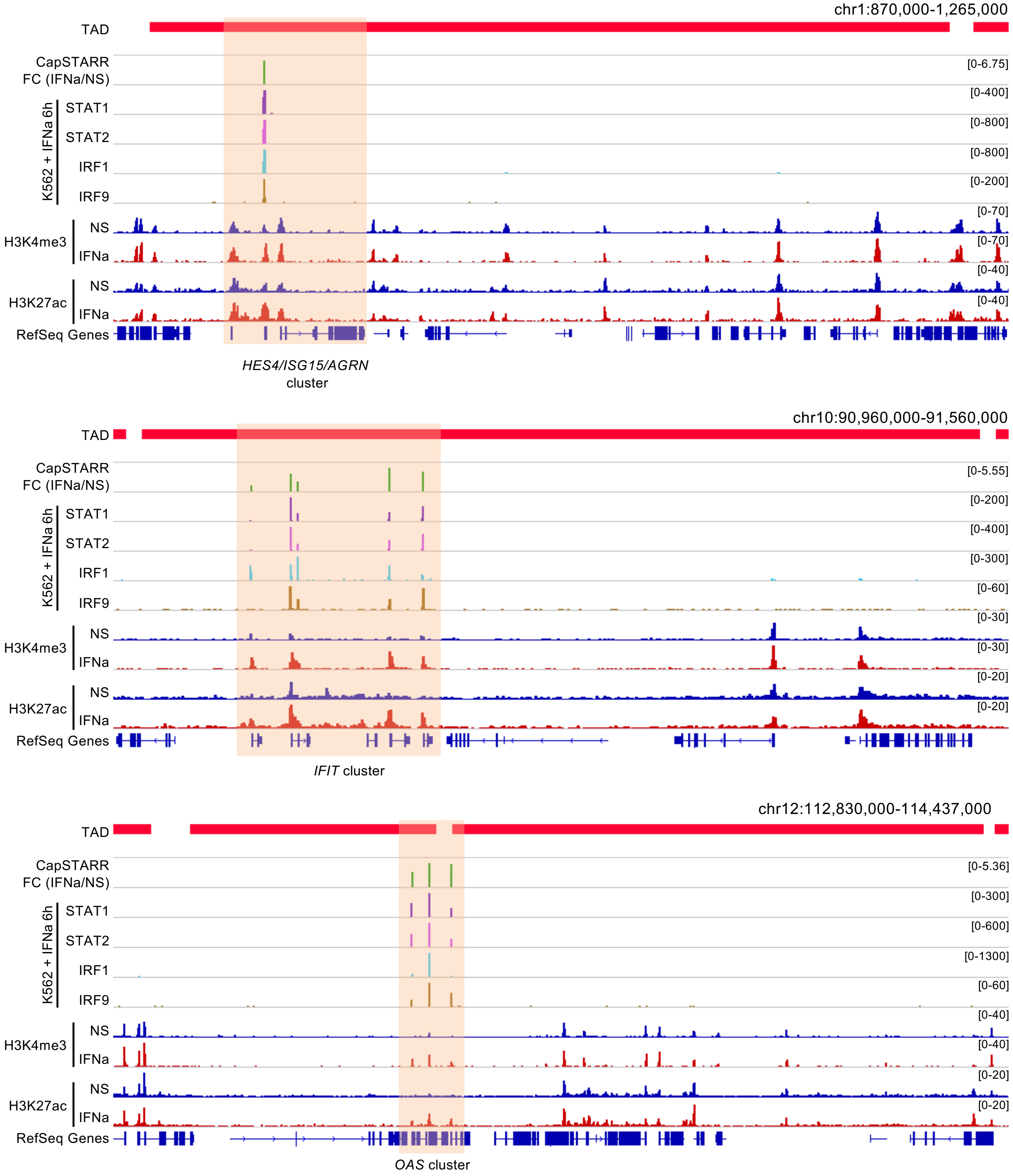

Supplementary Fig. 6 Genomic visualization of TADs harboring IFNa-induced clusters. Genomic tracks showing the TAD, the fold change of the CapSTARR-seq signal, the ChIP-seq signal for the indicated transcription factors after IFNa induction and the ChIP-seq signal for the histone modifications in H3K4me3 and H3K27ac in non-stimulated and IFNa-stimulated K562 cells. The cluster of *HES4/ISG15/AGRN* (upper panel), *IFIT* genes (middle panel) and *OAS* genes (lower panel) are highlighted.

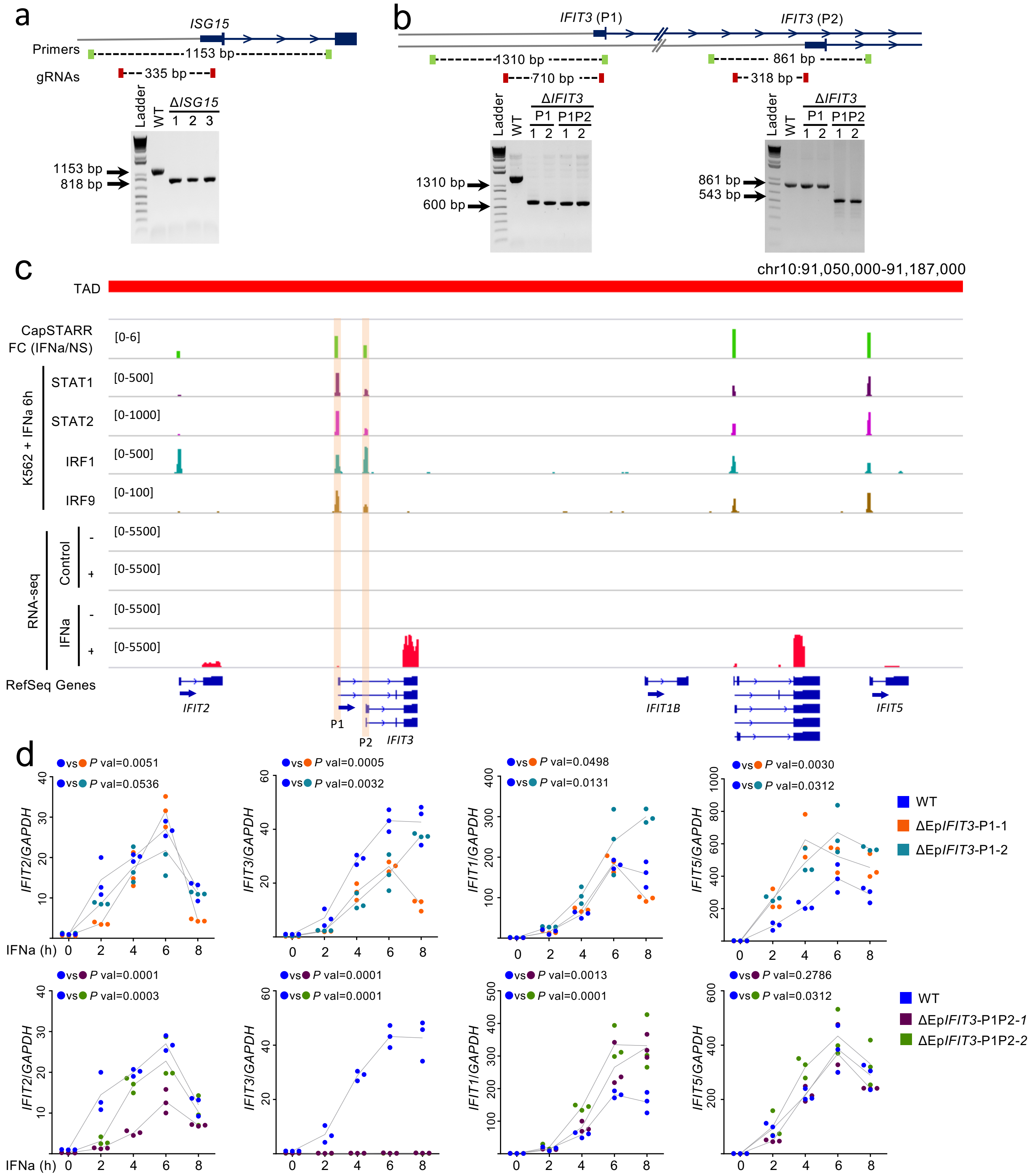

Supplementary Fig. 7 Genomic visualization and kinetic analysis of the IFNa induced clusters IFIT. a, b CRISPR deletion strategy of the Epromoter of ISG15 (**a**), and the two promoters of IFIT3 (**b**). Primers (green) and gRNAs (red) used, along with the agarose gel with the PCR products of the *wild-type* and the mutant clones are shown. Results are representative of at least three independent analyses. **c** Genomic tracks centered on the *IFIT* loci, with the TAD region, CapSTARR signal fold change (IFNa over non-stimulated), the ChIP-seq signal for the indicated transcription factors after IFNa induction and the RNA-seq signal. Positive and negative signs indicate the RNA-seq signal on the sense and anti-sense strands, respectively. The Epromoters of the *IFIT3* gene, P1 and P2, are highlighted. **d** qPCR analysis of gene expression of the IFIT cluster genes in K562 wild-type *versus* two ΔEp*IFIT3* clones only for the P1 Epromoter (n=3) (upper panels), and *versus* two ΔEp*IFIT3* clones for both P1 and P2 Epromoters (n=3) (bottom panels) in non-stimulated conditions or after the indicated times of IFNa stimulation. Values represent the relative expression levels as compared to the *wild-type* in non-stimulated conditions and normalized by the *GAPDH* housekeeping gene. Two-side ANOVA test was performed and *P* values between the *wild-type* and the mutant clone are indicated.

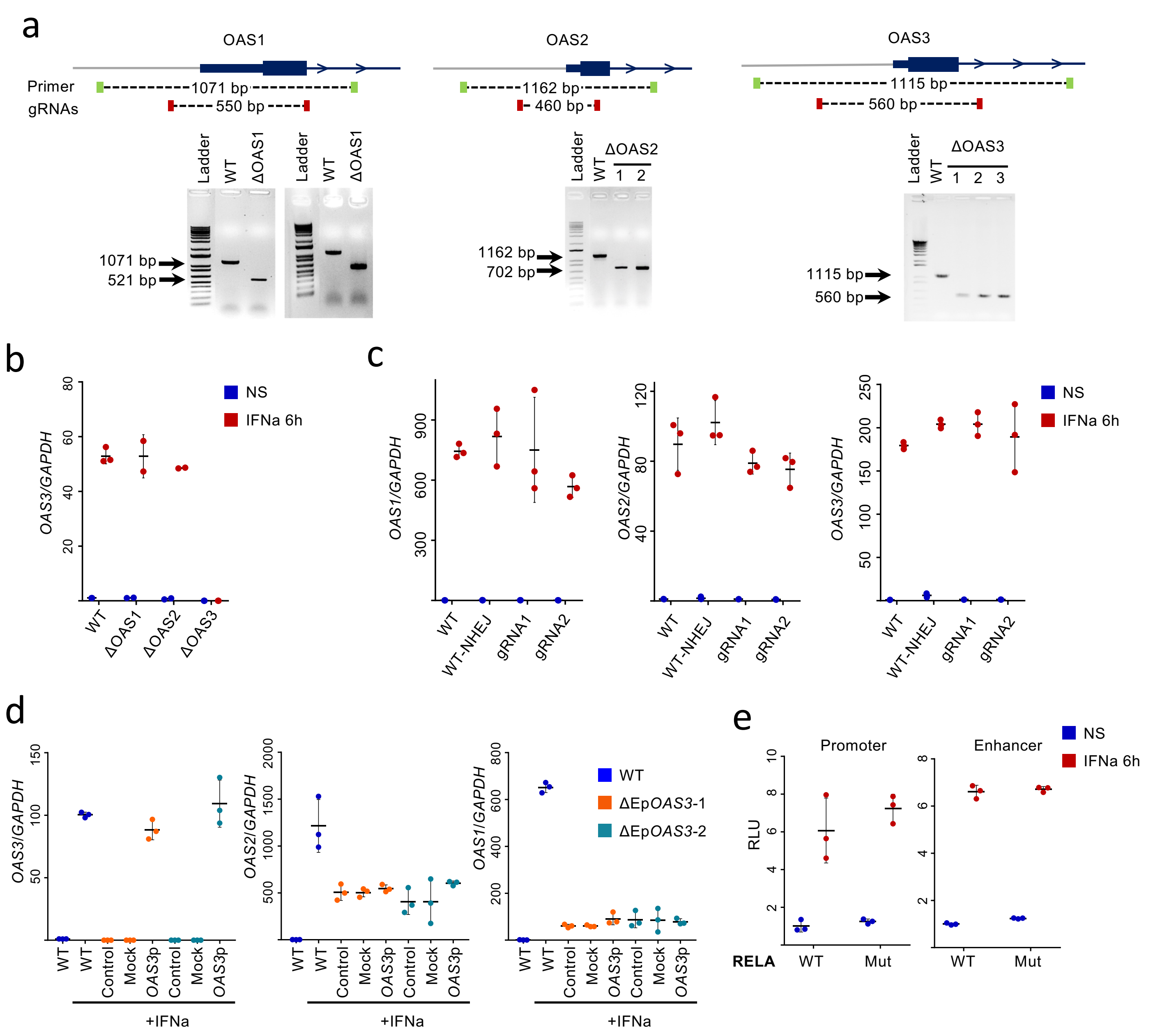

Supplementary Fig. 8 Gene expression of *OAS3* in the different mutants of the *OAS* cluster. a CRISPR deletion strategy of the Epromoter of *OAS1*, *OAS2*, and *OAS3*. Primers (green) and gRNAs (red) used, along with the agarose gel with the PCR products of the *wild-type* and the mutant clones are shown. Results are representative of at least three independent analyses. **b** qPCR analysis of gene expression of *OAS3* in K562 *wild-type* (n=3), ΔEp*OAS1* (n=2), ΔEp*OAS2* (n=2), and ΔEp*OAS3* (n=3) clones, in non-stimulated (blue) and IFNa-stimulated for 6 hours (red). Error bars indicate the media and the s.d. of independent experiments. No statistical difference was identified between *wild-type* and the ΔEp*OAS1 and* ΔEp*OAS2* clones (Two-side Student's *t* test). **c** qPCR analysis of the expression of the *OAS* genes in K562 *wild-type* cells (n=3), wild-type clone that had a non-homologous end-joint recombination (chr12:113,376,57, A base insertion) with the gRNA2 (n=3), the pool of *wild-type* transfected cells with either the gRNA1 (n=3) or gRNA2 (n=3) for the *OAS3*-CRISPR deletion, in non-stimulated (blue) and IFNa-stimulated (red) conditions. Error bars indicate the media and the s.d. of independent experiments. No statistical difference was identified (Two-side Student's *t* test). **d** *OAS3* expression rescue in ΔEp*OAS3* clones 1 and 2. qPCR analysis of the expression of the *OAS* genes in K562 *wild-type* cells (n=3), ΔEp*OAS3* clones non-transfected (control, n=3), transfected with GFP reporter gene (mock, n=3) and transfected with the *OAS3* gene expression vector for (*OAS3*p, n=3) in IFNa-stimulated conditions. Error bars indicate the media and s.d. of independent experiments. No statistical difference was observed in the expression of *OAS1* and *OAS2* between the control, the mock and the *OAS3*p for each clone (Two-side Student's *t* test). **e** Luciferase assay to quantify the promoter (left) and the enhancer (right) activity of the wild-type *OAS3* Epromoter or with the RELA mutation in non-stimulated (blue) or IFNa-stimulated (red) cells. Error bars indicate the media and s.d. of three independent experiments.

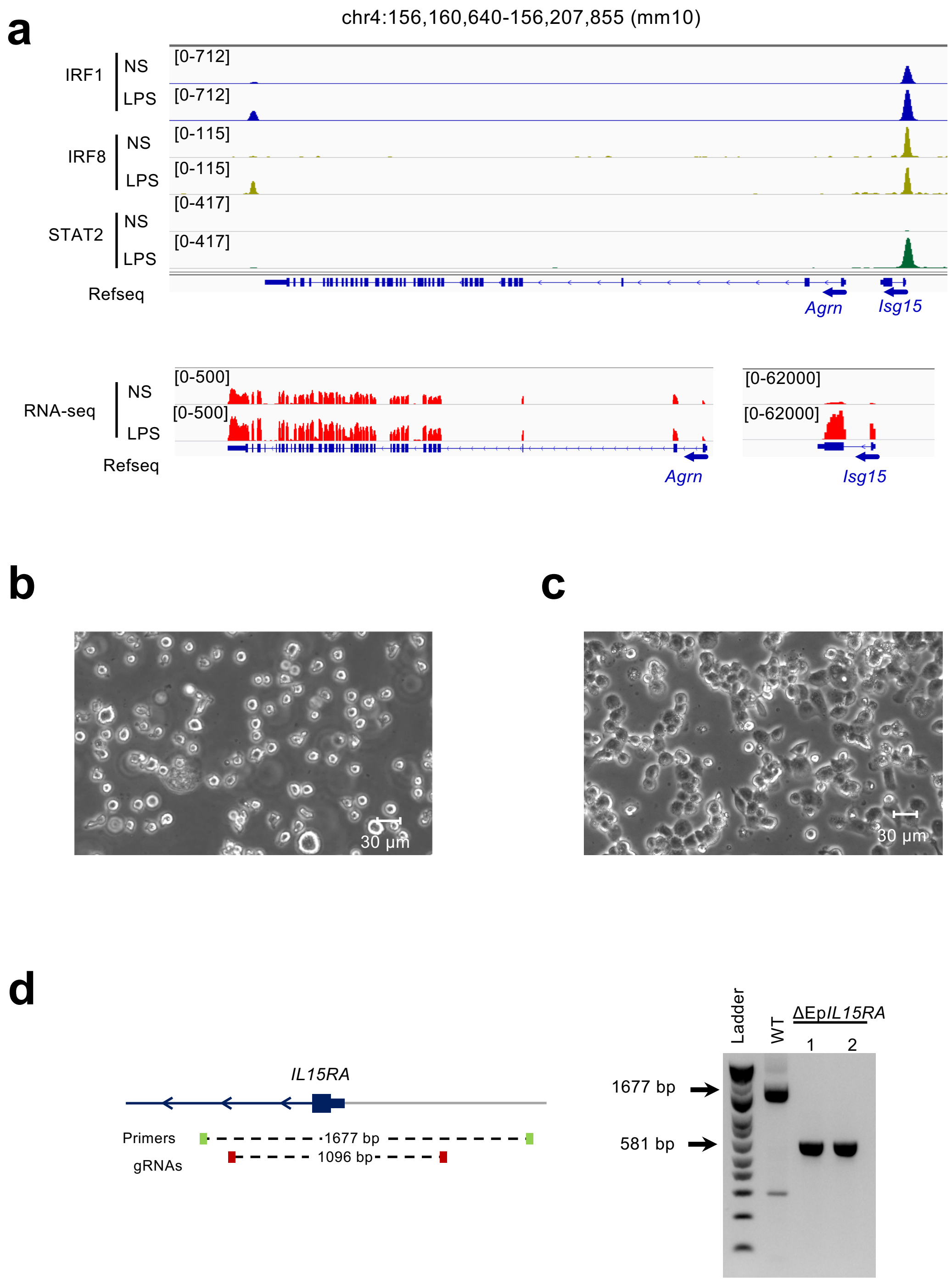

Supplementary Fig. 9 Macrophage stimulation by LPS. a Genomic tracks at the *Isg15* cluster showing the ChIP-seq signal of the transcription factors IRF1 (blue), IRF8 (light olive) and STAT2 (green), before, non stimulated (NS), and after 4 hours of lipopolysaccharide stimulation (LPS), and the RNA-seq (red) before and after the stimulation in the mouse macrophages (data taken from Mancino et al., 2015). *Isg15* and *Agrn* are significantly induced by LPS, but only the *Isg15* promoter bind the specific transcription factors. **b-c** Representative microscopy images showing changes in morphology of the *in vitro* differentiated THP-1 macrophages. THP-1 monocytes cell lines in suspension (**b**) and *in vitro* differentiated THP-1 macrophages. (**c**) upon 48 hours of treatment with PMA at 10 ng/mL. Differentiation was visually confirmed before each experiment. **d** Representative agarose gel electrophoresis of PCR products using primers (green) designed outside the deleted region (red) amplified on genomic DNA from the WT and mutants Δp*IL15RA* clones. The deletion of the *IL15RA* promoter was confirmed at least three times
