# Supplementary material for: Epromoters function as a hub to recruit key transcription factors required for the inflammatory response
Source: Nat Commun. 2021 Nov 18;12:6660. doi: 10.1038/s41467-021-26861-0 (PMC8602369; doi:10.1038/s41467-021-26861-0)
Supplement: Supplementary file 2 — Description of additional Supplementary File [file 41467_2021_26861_MOESM2_ESM.pdf]

Description of Additional Supplementary Files

File Name: Supplementary Data 1.
Description: The summary list of all RNA-seq and CapSTARR-seq data in non-stimulated (NS) and stimulated (IFNa) K562 cells.

File Name: Supplementary Data 2.
Description: The summary list of the three sets of IFNa-response loci in K562 cells.

File Name: Supplementary Data 3.
Description: The list of Gene Ontology (GO) term enrichment of the different sets of IFNa-response loci.

File Name: Supplementary Data 4.
Description: The list of all data resources used in this study.

File Name: Supplementary Data 5.
Description: The list of IFNaresponse gene clusters in K562.

File Name: Supplementary Data 6.
Description: The list of LPS induced gene clusters in mouse macrophages.

File Name: Supplementary Data 7.
Description: The list of all primers and gRNAs sequences used in this study.
