# Supplementary material for: Epromoters function as a hub to recruit key transcription factors required for the inflammatory response
Source: Nat Commun. 2021 Nov 18;12:6660. doi: 10.1038/s41467-021-26861-0 (PMC8602369; doi:10.1038/s41467-021-26861-0)
Supplement: Supplementary file 10 — Reporting Summary [file 41467_2021_26861_MOESM10_ESM.pdf]

nature portfolio

Corresponding author(s): Salvatore Spicuglia

Last updated by author(s): 20/09/2021

Reporting Summary

Nature Portfolio wishes to improve the reproducibility of the work that we publish. This form provides structure for consistency and transparency in reporting. For further information on Nature Portfolio policies, see our Editorial Policies and the Editorial Policy Checklist.

Statistics

For all statistical analyses, confirm that the following items are present in the figure legend, table legend, main text, or Methods section.

n/a	Confirmed	
[ ]	[x]	The exact sample size (*n*) for each experimental group/condition, given as a discrete number and unit of measurement
[ ]	[x]	A statement on whether measurements were taken from distinct samples or whether the same sample was measured repeatedly
[ ]	[x]	The statistical test(s) used AND whether they are one- or two-sided<br>*Only common tests should be described solely by name; describe more complex techniques in the Methods section.*
[x]	[ ]	A description of all covariates tested
[ ]	[x]	A description of any assumptions or corrections, such as tests of normality and adjustment for multiple comparisons
[ ]	[x]	A full description of the statistical parameters including central tendency (e.g. means) or other basic estimates (e.g. regression coefficient) AND variation (e.g. standard deviation) or associated estimates of uncertainty (e.g. confidence intervals)
[ ]	[x]	For null hypothesis testing, the test statistic (e.g. *F*, *t*, *r*) with confidence intervals, effect sizes, degrees of freedom and *P* value noted<br>*Give P values as exact values whenever suitable.*
[x]	[ ]	For Bayesian analysis, information on the choice of priors and Markov chain Monte Carlo settings
[x]	[ ]	For hierarchical and complex designs, identification of the appropriate level for tests and full reporting of outcomes
[x]	[ ]	Estimates of effect sizes (e.g. Cohen's *d*, Pearson's *r*), indicating how they were calculated

Our web collection on statistics for biologists contains articles on many of the points above.

Software and code

Policy information about availability of computer code

Data collection	No software was used for data collection
Data analysis	All custom scripts used in this study are available at GitHub (https://github.com/Spicuglia-Lab/IFN-Data-Analysis) and archived at https://doi.org/10.5281/zenodo.5507612. Other software used in this study are: Bowtie (v2.3.4.3), STAR aligner (v2.4.2a), Sickle (v1.33), RSeQC program suite (v2.6.4), DESeq2 (v1.6.3), SamTools (v0.1.9, v1.13), BedTools (v2.17.0, v2.28.0), OLOGRAM tool (V1), DeepTools (v3.2.1), Macs2 (v2.2.7.1), SeqPlots tool (v1), IGV genome browser (v2.11.1), GREAT (v4.0.4), HOMER (V2), CrispRGold (v1.1), webtool CRISPRdirect (V1), GraphPad (7.0), R version (4.1.0).

For manuscripts utilizing custom algorithms or software that are central to the research but not yet described in published literature, software must be made available to editors and reviewers. We strongly encourage code deposition in a community repository (e.g. GitHub). See the Nature Portfolio guidelines for submitting code & software for further information.

Data

Policy information about availability of data

All manuscripts must include a data availability statement. This statement should provide the following information, where applicable:

- Accession codes, unique identifiers, or web links for publicly available datasets
- A description of any restrictions on data availability
- For clinical datasets or third party data, please ensure that the statement adheres to our policy

The RNA-Seq, CapSTARR-seq and ChIP-Seq data generated in this study have been submitted to the Gene Expression Omnibus (GEO) under the accession code GSE159462 (https://www.ncbi.nlm.nih.gov/geo/query/acc.cgi?acc=GSE159462). Public datasets can be found under accession codes: ENCSR000FAU (https://www.encodeproject.org/experiments/ENCSR000FAU/), ENCSR000FBC (https://www.encodeproject.org/experiments/ENCSR000FBC/), ENCSR000EGL (https://

www.encodeproject.org/experiments/ENCSR000EGL/), ENCSR669GJD (https://www.encodeproject.org/experiments/ENCSR669GJD/), GSE183296 (https://www.ncbi.nlm.nih.gov/geo/query/acc.cgi?acc=GSE83296), GSE63525 (https://www.ncbi.nlm.nih.gov/geo/query/acc.cgi?acc=GSE63525), GSE56123 (https://www.ncbi.nlm.nih.gov/geo/query/acc.cgi?acc=GSE56123), GSE96800 (https://www.ncbi.nlm.nih.gov/geo/query/acc.cgi?acc=GSE96800), GSE89663 (https://www.ncbi.nlm.nih.gov/geo/query/acc.cgi?acc=GSE89663). All generated and publicly available datasets are listed in the Supplementary Data 4. Public database used in this study are: Jaspar 2020 http://genome.ucsc.edu/cgi-bin/hgTrackUi?hgsid=1169499883_qhnKn3jHG401qgfVk2LXUvfvZ2rU&db=hg38&c=chr19&g=hub_186875_JASPAR2020_TFBS_hg38); Interferome database (http://www.interferome.org/interferome/home.jspx). A reporting summary for this article is available as a Supplementary Information file. Source data are provided as a Source Data file.

Field-specific reporting

Please select the one below that is the best fit for your research. If you are not sure, read the appropriate sections before making your selection.

☐ Life sciences ☐ Behavioural & social sciences ☐ Ecological, evolutionary & environmental sciences

For a reference copy of the document with all sections, see nature.com/documents/nr-reporting-summary-flat.pdf

Life sciences study design

All studies must disclose on these points even when the disclosure is negative.

Sample size	Samples size was not determined by calculations. Each experiment was performed in triplicate. the number of CRISPR edited clones was determined by the screening process
Data exclusions	No data was excluded
Replication	The reproducibility was achieved by performing a minimum of three replicates for each experiment
Randomization	Randomization was not applied in our study as we did not had a clinical research involving treatments.
Blinding	Due to the experimental design and goals of our article sample blinding was not neccessary.

Behavioural & social sciences study design

All studies must disclose on these points even when the disclosure is negative.

Study description	*Briefly describe the study type including whether data are quantitative, qualitative, or mixed-methods (e.g. qualitative cross-sectional, quantitative experimental, mixed-methods case study).*
Research sample	*State the research sample (e.g. Harvard university undergraduates, villagers in rural India) and provide relevant demographic information (e.g. age, sex) and indicate whether the sample is representative. Provide a rationale for the study sample chosen. For studies involving existing datasets, please describe the dataset and source.*
Sampling strategy	*Describe the sampling procedure (e.g. random, snowball, stratified, convenience). Describe the statistical methods that were used to predetermine sample size OR if no sample-size calculation was performed, describe how sample sizes were chosen and provide a rationale for why these sample sizes are sufficient. For qualitative data, please indicate whether data saturation was considered, and what criteria were used to decide that no further sampling was needed.*
Data collection	*Provide details about the data collection procedure, including the instruments or devices used to record the data (e.g. pen and paper, computer, eye tracker, video or audio equipment) whether anyone was present besides the participant(s) and the researcher, and whether the researcher was blind to experimental condition and/or the study hypothesis during data collection.*
Timing	*Indicate the start and stop dates of data collection. If there is a gap between collection periods, state the dates for each sample cohort.*
Data exclusions	*If no data were excluded from the analyses, state so OR if data were excluded, provide the exact number of exclusions and the rationale behind them, indicating whether exclusion criteria were pre-established.*
Non-participation	*State how many participants dropped out/declined participation and the reason(s) given OR provide response rate OR state that no participants dropped out/declined participation.*
Randomization	*If participants were not allocated into experimental groups, state so OR describe how participants were allocated to groups, and if allocation was not random, describe how covariates were controlled.*

Ecological, evolutionary & environmental sciences study design

All studies must disclose on these points even when the disclosure is negative.

Study description	*Briefly describe the study. For quantitative data include treatment factors and interactions, design structure (e.g. factorial, nested, hierarchical), nature and number of experimental units and replicates.*
Research sample	*Describe the research sample (e.g. a group of tagged Passer domesticus, all Stenocereus thurberi within Organ Pipe Cactus National Monument), and provide a rationale for the sample choice. When relevant, describe the organism taxa, source, sex, age range and any manipulations. State what population the sample is meant to represent when applicable. For studies involving existing datasets, describe the data and its source.*
Sampling strategy	*Note the sampling procedure. Describe the statistical methods that were used to predetermine sample size OR if no sample-size calculation was performed, describe how sample sizes were chosen and provide a rationale for why these sample sizes are sufficient.*
Data collection	*Describe the data collection procedure, including who recorded the data and how.*
Timing and spatial scale	*Indicate the start and stop dates of data collection, noting the frequency and periodicity of sampling and providing a rationale for these choices. If there is a gap between collection periods, state the dates for each sample cohort. Specify the spatial scale from which the data are taken*
Data exclusions	*If no data were excluded from the analyses, state so OR if data were excluded, describe the exclusions and the rationale behind them, indicating whether exclusion criteria were pre-established.*
Reproducibility	*Describe the measures taken to verify the reproducibility of experimental findings. For each experiment, note whether any attempts to repeat the experiment failed OR state that all attempts to repeat the experiment were successful.*
Randomization	*Describe how samples/organisms/participants were allocated into groups. If allocation was not random, describe how covariates were controlled. If this is not relevant to your study, explain why.*
Blinding	*Describe the extent of blinding used during data acquisition and analysis. If blinding was not possible, describe why OR explain why blinding was not relevant to your study.*

Did the study involve field work? ☐ Yes ☐ No

Field work, collection and transport

Field conditions	*Describe the study conditions for field work, providing relevant parameters (e.g. temperature, rainfall).*
Location	*State the location of the sampling or experiment, providing relevant parameters (e.g. latitude and longitude, elevation, water depth).*
Access & import/export	*Describe the efforts you have made to access habitats and to collect and import/export your samples in a responsible manner and in compliance with local, national and international laws, noting any permits that were obtained (give the name of the issuing authority, the date of issue, and any identifying information).*
Disturbance	*Describe any disturbance caused by the study and how it was minimized.*

Reporting for specific materials, systems and methods

We require information from authors about some types of materials, experimental systems and methods used in many studies. Here, indicate whether each material, system or method listed is relevant to your study. If you are not sure if a list item applies to your research, read the appropriate section before selecting a response.

Materials & experimental systems

n/a	Involved in the study	
☐	☒	Antibodies
☐	☒	Eukaryotic cell lines
☒	☐	Palaeontology and archaeology
☒	☐	Animals and other organisms
☒	☐	Human research participants
☒	☐	Clinical data
☒	☐	Dual use research of concern

Methods

n/a	Involved in the study	
☐	☒	ChIP-seq
☒	☐	Flow cytometry
☒	☐	MRI-based neuroimaging

Antibodies

Antibodies used	IRF9 Rabbit IgG, clone D2T8M. Cell signal #766845.<br>H3K27ac Rabbit mAB, Diagenode #C15210016<br>H3K4me3 Rabbit polyclonal AB. Diagenode #C15410003<br>H3K4me1 Rabbit polyclonal AB. Diagenode #C15410194
Validation	IRF9 antibody has been validated SimpleChIP® Enzymatic Chromatin IP Kits by Cell Signal<br>H3K27ac, H3K4me3 and H3K4me1 were validated by ChIP-seq by Diagenode<br>All antibodies were validated in the lab by ChIP analyses using known potive and negative conrol

Eukaryotic cell lines

Policy information about cell lines

Cell line source(s)	K562 (CCL-243) cell line was obtained from ATCC<br>THP-1 (ACC-16) was obtained by DSMZ
Authentication	The cell lines were not authenticated
Mycoplasma contamination	Negative to mycoplasma contamination
Commonly misidentified lines<br>(See ICLAC register)	No commonly misidentified lines were used

ChIP-seq

Data deposition

☒ Confirm that both raw and final processed data have been deposited in a public database such as GEO.

☒ Confirm that you have deposited or provided access to graph files (e.g. BED files) for the called peaks.

Data access links<br>*May remain private before publication.*	No commonly misidentified lines were used
Files in database submission	Chip-seq IRF9 K562 + IFNa 6h<br>Chip-seq H3K27ac K562<br>Chip-seq H3K27ac K562 + IFNa 6h<br>Chip-seq H3K4me3 K562<br>Chip-seq H3K4me3 K562 + IFNa 6h<br>Chip-seq H3K4me1 K562<br>Chip-seq H3K4me1 K562 + IFNa 6h
Genome browser session<br>(e.g. UCSC)	no longer applicable

Methodology

Replicates	Single replicate ChIP
Sequencing depth	38.9 million reads paired-end 50+30 bp. 36.7 million reads after sickle trimming. 27.8 million uniquely mapped reads. Fragment lenght 215 bp.
Antibodies	IRF9 Rabbit IgG, clone D2T8M. Cell signal #766845.<br>H3K27ac Rabbit mAB, Diagenode #C15210016<br>H3K4me3 Rabbit polyclonal AB. Diagenode #C15410003<br>H3K4me1 Rabbit polyclonal AB. Diagenode #C15410194
Peak calling parameters	Reads were aligned using bowtie2 on hg19 assembly with default parameters and further filtered using samtools view with "-q 30" parameter. Peaks were called using MACS2 with "--gs-size hs" parameter using input.
Data quality	Peaks were called with a 5% FDR threshold
Software	Base-calling with bcl2fastq v2.19.0<br>Trimming with sickle v1.33 and "-t-sanger -q 20" arguments<br>Alignment on hg19 genome with bowtie v2.4.2<br>Filtering on mapping quality using samtools v1.1 and "view -q 30" argument<br>Peaks were called using Macs2 v2.1.1<br>Bigwig files were produced using DeepTools bamCoverage using RPKM normalization.
